# PI 3 Kinase Related Kinases-Independent Proteolysis of BRCA1 Regulates Rad51 Recruitment during Genotoxic Stress in Human Cells

**DOI:** 10.1371/journal.pone.0014027

**Published:** 2010-11-17

**Authors:** Ian Hammond-Martel, Helen Pak, Helen Yu, Raphael Rouget, Andrew A. Horwitz, Jeffrey D. Parvin, Elliot A. Drobetsky, El Bachir Affar

**Affiliations:** 1 Department of Medicine, Maisonneuve-Rosemont Hospital Research Center, University of Montréal, Montréal, Québec, Canada; 2 Department of Pathology, Harvard Medical School and Brigham and Women's Hospital, Boston, Massachusetts, United States of America; Tulane University Health Sciences Center, United States of America

## Abstract

**Background:**

The function of BRCA1 in response to ionizing radiation, which directly generates DNA double strand breaks, has been extensively characterized. However previous investigations have produced conflicting data on mutagens that initially induce other classes of DNA adducts. Because of the fundamental and clinical importance of understanding BRCA1 function, we sought to rigorously evaluate the role of this tumor suppressor in response to diverse forms of genotoxic stress.

**Methodology/Principal Findings:**

We investigated BRCA1 stability and localization in various human cells treated with model mutagens that trigger different DNA damage signaling pathways. We established that, unlike ionizing radiation, either UVC or methylmethanesulfonate (MMS) (generating bulky DNA adducts or alkylated bases respectively) induces a transient downregulation of BRCA1 protein which is neither prevented nor enhanced by inhibition of PIKKs. Moreover, we found that the proteasome mediates early degradation of BRCA1, BARD1, BACH1, and Rad52 implying that critical components of the homologous recombinaion machinery need to be functionally abrogated as part of the early response to UV or MMS. Significantly, we found that inhibition of BRCA1/BARD1 downregulation is accompanied by the unscheduled recruitment of both proteins to chromatin along with Rad51. Consistently, treatment of cells with MMS engendered complete disassembly of Rad51 from pre-formed ionizing radiation-induced foci. Following the initial phase of BRCA1/BARD1 downregulation, we found that the recovery of these proteins in foci coincides with the formation of RPA and Rad51 foci. This indicates that homologous recombination is reactivated at later stage of the cellular response to MMS, most likely to repair DSBs generated by replication blocks.

**Conclusion/Significance:**

Taken together our results demonstrate that (i) the stabilities of BRCA1/BARD1 complexes are regulated in a mutagen-specific manner, and (ii) indicate the existence of mechanisms that may be required to prevent the simultaneous recruitment of conflicting signaling pathways to sites of DNA damage.

## Introduction

Germline mutations in *BRCA1* cause extremely high predisposition to breast and ovarian cancers. BRCA1 is a large protein with a well-established modular structure. It contains two BRCT domains at the C-terminus, i.e., phospho-peptide binding modules also carried by several proteins involved in the DNA damage response. The N-terminus of BRCA1 is characterized by the presence of a Ring finger domain conferring ubiquitin ligase activity via stable complex formation with another Ring finger protein, BRCA1-associated RING domain 1 (BARD1). Although the precise role(s) of BRCA1/BARD1 in tumor suppression have not been fully established, ample evidence indicates that this heterodimer is required to maintain genomic stability following DNA damage (see reviews [1], [2]). During periods of genotoxic stress BRCA1 is rapidly phosphorylated and thus activated by the primary responders Ataxia-Telangiectasia-Mutated kinase (ATM) or ATM- and Rad3-Related kinase (ATR), which in turn promotes cellular recovery through induction of DNA damage checkpoints [3], [4], [5], [6], [7]. Moreover, recent studies indicate that BRCA1/BARD1 selectively associates with several components of the DNA damage response forming mutually exclusive complexes. Indeed, through the BRCT domain, BRCA1/BARD1 interacts with either Abraxas, BACH1, or CtIP, along with other distinct cofactors, to form multiprotein complexes termed A, B, and C, respectively. These complexes play important roles in the DNA damage response by exerting specific although overlapping functions in cell cycle arrest and DNA repair [2], [8].

The role of BRCA1 has been studied mostly in the context of ionizing radiation (IR), which directly induces highly genotoxic DNA double-strand breaks (DSBs). Following exposure to IR, several proteins are rapidly recruited to DSB sites to form IR-Induced Foci (IRIF). IRIFs are characterized by ATM-mediated phosphorylation of the histone variant H2AX (γH2AX) [9], which is required for the subsequent highly coordinated assembly of checkpoint/repair proteins. The precise mechanism of IRIF formation is not completely understood, although recent studies have shed light on the dynamics and orchestration of this process. The DNA damage mediator MDC1 promotes recruitment of the E3 ligases RNF8 and RNF168 that ubiquitinate specific substrates including histones. These events are required for interaction with the ubiquitin binding protein RAP80, which then recruits additional factors including BRCA1 and BARD1. At the IRIF, BRCA1/BARD1 in turn attracts other proteins such as Rad51 and BRCA2 that mediate cell cycle checkpoints and DNA repair (reviewed recently in [2], [8], [10]).

In contrast to the situation for IR, the manner in which BRCA1 responds to genotoxic agents that do not directly induce DSBs is poorly understood. BRCA1 was initially reported to be rapidly dispersed from constitutive foci following treatment with various mutagens [11], [12]. These normally-occurring BRCA1 foci (constitutive foci as opposed to IRIF) contain a substantial pool of BRCA1 and are found in ∼ 40–70% of the cells [11], [13], [14]. Little is known about the significance of these foci in unstressed cells, although one recent study suggests that these might be associated with replication of pericentric heterochromatin [15]. Moreover, the manner in which BRCA1 dispersion occurs, and the significance of this event, remain to be elucidated. In particular it has been unclear whether there might be a relationship between this dispersion and changes in protein stability during DNA damage. Although it is often assumed that the phosphorylation state, rather than absolute levels, of BRCA1 changes in response to DNA damage [3], [5], [6], [7], [16], [17], [18], some studies reported that BRCA1 and/or BARD1 are upregulated following treatment with UV or the topoisomerase II inhibitor doxorubicin [19], [20], [21], [22], [23]. In sharp contrast, other investigations reported that these proteins are downregulated following treatment with the same agents [24], [25]. Recently, it was shown that BARD1 is downregulated in a proteasome-dependent manner following treatment with an extremely cytotoxic dose of UV (70 J/m^2^ ) that induces substantial levels of apoptosis [26]. However, under the same conditions, significant changes in BRCA1 levels were not consistently observed. It is also critical to emphasize that BRCA1 was shown to be rapidly cleaved during apoptosis induced by high dose UV, thereby possibly accounting for the aformentioned inconsistency [27], [28], [29]. BRCA1 protein levels and subnuclear localization have also been investigated following treatment of cells with DNA alkylating agents. One study reported that this protein accumulates in nuclear foci following treatment with methylmethanesulfonate (MMS) [30], whereas another showed that BRCA1 is actually downregulated by methyl methanethiosulfonate [31]. In summary, it is not yet clear how BRCA1/BARD1 stability and subcellular localization are regulated in response to diverse classes of DNA adducts, other than DSBs, which trigger unique though overlapping signaling pathways.

Defining how BRCA1 participates in the DNA damage response is of a major importance not only for understanding breast and ovarian cancer development, but also towards helping to improve current cancer therapeutic protocols. For example several promising clinical trials are based on the use of inhibitors of the DNA damage-responsive enzyme PARP1 as a means to selectively target BRCA1-deficient tumor cells [32], [33]. In view of the importance of BRCA1 in cancer development and treatment, and the conflicting data in the literature as cited above, we were prompted to carefully evaluate BRCA1 stability and localization in the cellular response to diverse-acting DNA damaging agents. We conclusively demonstrate that BRCA1 stability is regulated in a mutagen-specific manner. Indeed, in the early response to UV and MMS, but not to IR, dispersion of BRCA1/BARD1 from nuclear foci is accompanied by ubiquitin-mediated degradation of both tumor suppressors. Significantly, BRCA1 downregulation does not involve the major DNA damage-activated PI 3 Kinase Related Kinases (PIKK) or Mitogen-Activated Protein Kinase (MAPK) pathways, suggesting that other yet to be identified signaling mechanisms regulate BRCA1 stability/function following DNA damage. Furthermore, we reveal that BACH1 and Rad52 are also degraded in a proteasome-dependent manner indicating that critical components of the homologous recombination (HR) machinery are selectively targeted for degradation. Finally, data is provided suggesting that DNA damage signaling pathways might need to be coordinated in order to forestall the untimely recruitment of potentially conflicting DNA damage responses.

## Results

### BRCA1 is downregulated in response to UVC or MMS, but not IR

**Figure 1:**
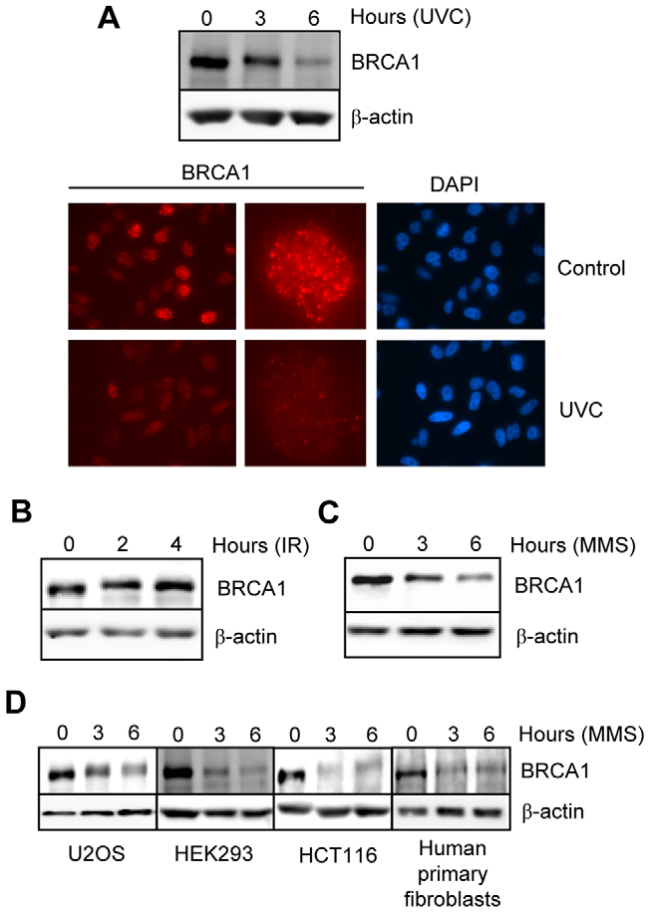
Downregulation of BRCA1 protein during genotoxic stress. A) *Top*, BRCA1 expression in HeLa cells treated with UVC (30 J/m^2^) was detected by immunoblotting after harvesting at the indicated times. *Bottom*, immunostaining of BRCA1 at 4 hrs post-treatment. DNA was counterstained with DAPI. B) BRCA1 levels in HeLa cells treated with IR (10 Gy) for the indicated times. C) BRCA1 levels in HeLa cells treated with the DNA alkylating agent, methylmethanesulfonate (MMS, 200 µM) for the indicated times. D) BRCA1 levels in various cell types treated with 200 µM MMS for the indicated times. All immunoblottings were conducted using total cell extracts. β-actin was detected to ensure equal protein loading.

Towards understanding the mechanisms that coordinate regulation of BRCA1 stability and localization following genotoxic stress, we initially treated HeLa cells with 30 J/m^2^ of 254-nm UV (UVC) which induces rapid ATR-dependent phosphorylation of BRCA1 [3], [16]. Using an antibody recognizing the N-terminal region of BRCA1, we found that UVC induced a substantial decrease in levels of this protein at 3 hrs post-treatment, which became more marked by 6 hrs (Fig. 1A, top panel). Of note, this occurred simultaneously with depletion of BRCA1 from nuclear foci (Fig. 1A. bottom panel). Thus, the previously described phenomenon of BRCA1 “dispersion” from constitutive foci after UVC irradiation [11], [12] appears to be associated with actual depletion of the protein. Interestingly, IR treatment which has been shown to result in early dispersion of BRCA1 from constitutive foci [11], did not significantly affect BRCA1 protein levels (Fig. 1B). We also conducted immunoblotting with other anti-BRCA1 antibodies that map to the middle and C-terminal regions and found that in each case a substantial fraction of the protein is downregulated post-UVC (Fig.S1). It is important to emphasize that BRCA1 is downregulated following treatment with doses as low as 10 J/m^2^ of UVC (Fig.S2). Next, in investigating an additional diverse-acting genotoxin, we revealed that BRCA1 is downregulated in a dose-dependent manner following treatment with the DNA alkylating agent MMS (Fig. 1C and Fig.S3). The above data demonstrate that the control of BRCA1 stability varies significantly in a mutagen-specific manner. We also show (Fig. 1D) that BRCA1 downregulation (i) is not cell-type specific, as it occurs in various tumor cell lines and moreover (ii) was observed in primary human fibroblasts, revealing that the downregulation is not specific to transformed or tumor-derived cells.

### BRCA1 downregulation is independent of apoptosis and is reversible

**Figure 2:**
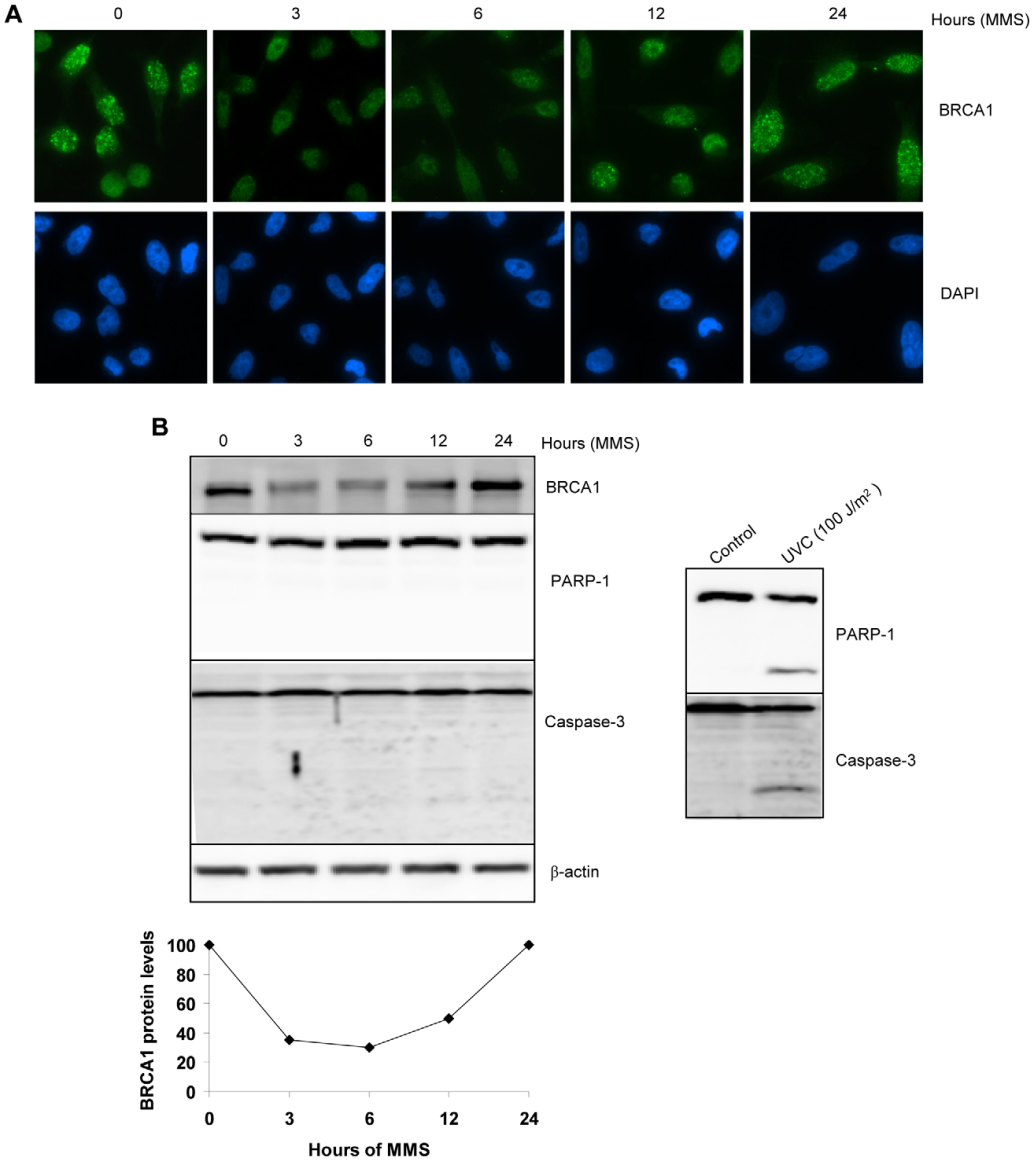
BRCA1 downregulation is independent of apoptosis and is reversible. A) Immunostaining of BRCA1 in HeLa cells treated with 200 µM MMS. Cells were harvested at 3 and 6 hrs or changed to MMS free medium for the later times. The nuclei were counterstained with DAPI. B) *Top left*, immunoblotting of BRCA1 and apoptosis markers, PARP-1 and Caspase-3, in HeLa cells treated as indicated above. *Bottom left*, BRCA1 band intensity was quantified and data are expressed as percentage of untreated cells. *Right*, immunoblotting for PARP-1 and Caspase-3 following treatment with high dose of UVC (100 J/m^2^).

To determine whether DNA damage-induced BRCA1 downregulation might be a consequence of cell death, HeLa cells were treated with 200 µM of MMS and harvested at various time points for immunostaining (Fig. 2A). BRCA1 protein exhibited maximal decrease at 3–6 hrs followed by its reappearance (reaching nearly 100% of basal levels) by 24 hrs post-treatment indicating that this decrease is transient. Under the above MMS treatment conditions, we did not observe cell death as indicated by the absence of any nuclear condensation typical of apoptosis (see nuclear staining by DAPI). Consistently, immunoblotting experiments also revealed a transient downregulation of BRCA1 (Fig. 2B, top panel). Densitometry quantification of BRCA1 protein levels confirmed these results (Fig. 2B, low panel). In addition, no cleavage of either PARP-1 or Caspase-3, two hallmarks of apoptosis, were detected in MMS-treated cells (Fig. 2B), and moreover no change in cell viability was observed during the treatments (∼100% viability at all time points as determined by trypan blue exclusion assay). Of note, to ensure that we were able to actually detect apoptosis in our experimental system, we treated cells with UVC (100 J/m^2^), and found that this highly toxic dose induced substantial apoptotic cleavage of Caspase-3 or PARP-1 after only 6 hrs post-treatment (Fig. 2B, right panel). The above results indicate that downregulation of BRCA1 is not a consequence of apoptosis, suggesting that a unique signaling process may underlie the temporal and spatial regulation of this protein.

### BRCA1 downregulation occurs in S and G2 phases of the cell cycle

**Figure 3:**
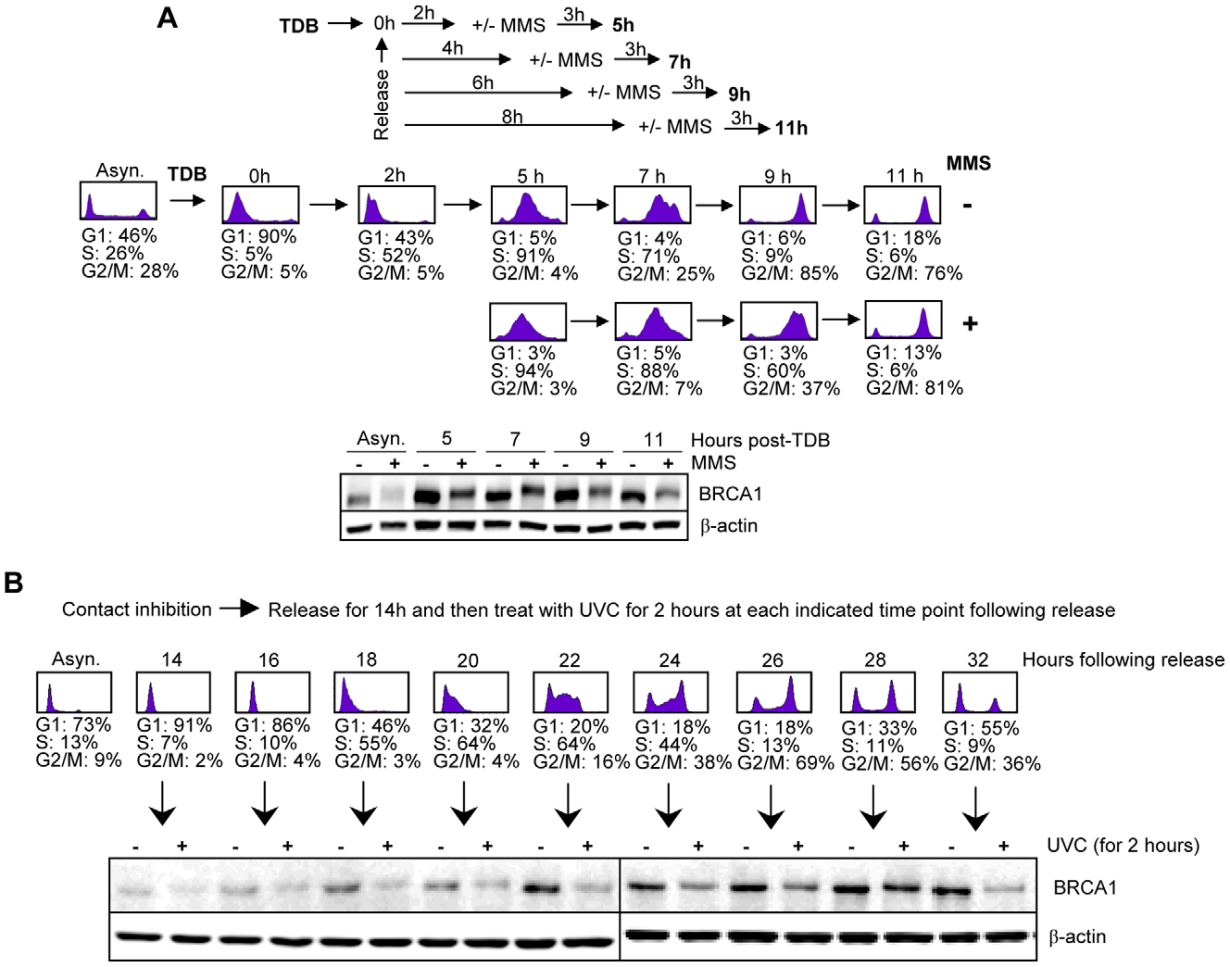
Downregulation of BRCA1 occurs independently of the cell cycle phases. A) Synchronized HeLa cells, using a thymidine double block (TDB) method, were treated with 200 µM MMS for 3 hrs at various time points post-release. Cell cycle analysis (top panel) and immunoblotting (low panel) were conducted at the indicated time points. B) Downregulation of BRCA1 during cell cycle progression in primary cells. *Top*, human primary fibroblasts were synchronized in G0/G1 by contact inhibition and were released by replating at low density. *Bottom*, following UVC (30 J/m^2^) treatment for the last 2 hrs, cell were harvested at the indicated times for immunoblotting. β-actin immunodetection was used as loading control.

Since (i) downregulation of BRCA1 after DNA damage is partial (Fig. 2), suggesting that this process might be specific to a distinct cell population, and (ii) BRCA1 is known to be expressed primarily during S and G2 phases [34], we evaluated whether DNA damage-induced BRCA1 downregulation might be triggered in a cell cycle-specific manner. HeLa cells were synchronized at the G1/S border using thymidine double block and treated with MMS for 3 hrs at different times post-release. Cell cycle profiles with or without MMS exposure reveal that more than 90% of the cells were in S phase at 5 hrs, and ∼80% in G2 at 11 hrs (Fig. 3A top panel). In accord with previous studies [34], BRCA1 protein levels were dramatically increased in S phase-enriched populations (Fig. 3A bottom panel, compare 5 hrs versus Asyn). We found that BRCA1 was downregulated at all time points examined after MMS treatment. Since the G2 population is not highly enriched under thymidine block (i.e., contaminated with S phase cells), we synchronized cells using other methods. G2 cells were highly purified (∼95%) after 16 hrs by pre-treatment with the mitotic inhibitor nocodazole in conjunction with mitotic shake-off to remove M cells (Fig.S4 left panel). G2 cells treated with MMS exhibited substantial downregulation of BRCA1 at 3 and 6 hrs (Fig.S4 right panel). We also synchronized primary human foreskin fibroblasts in G0 through a physiological process, i.e., contact inhibition, followed by release for various time points to allow progression through the cell cycle (Fig. 3B). We found that following UVC treatment, at any time during cell cycle progression up to 32 hr, BRCA1 is downregulated (Fig. 3B). The above data taken together conclusively demonstrate that the primary signal triggering BRCA1 downregulation during periods of genotoxic stress is not dependent upon cell cycle as might be expected *a priori*.

### The PI3 kinase related kinases (PIKKs) family members ATM, ATR and DNA-PK, and the canonical MAPKs, are not required for signaling BRCA1 downregulation following DNA damage

**Figure 4:**
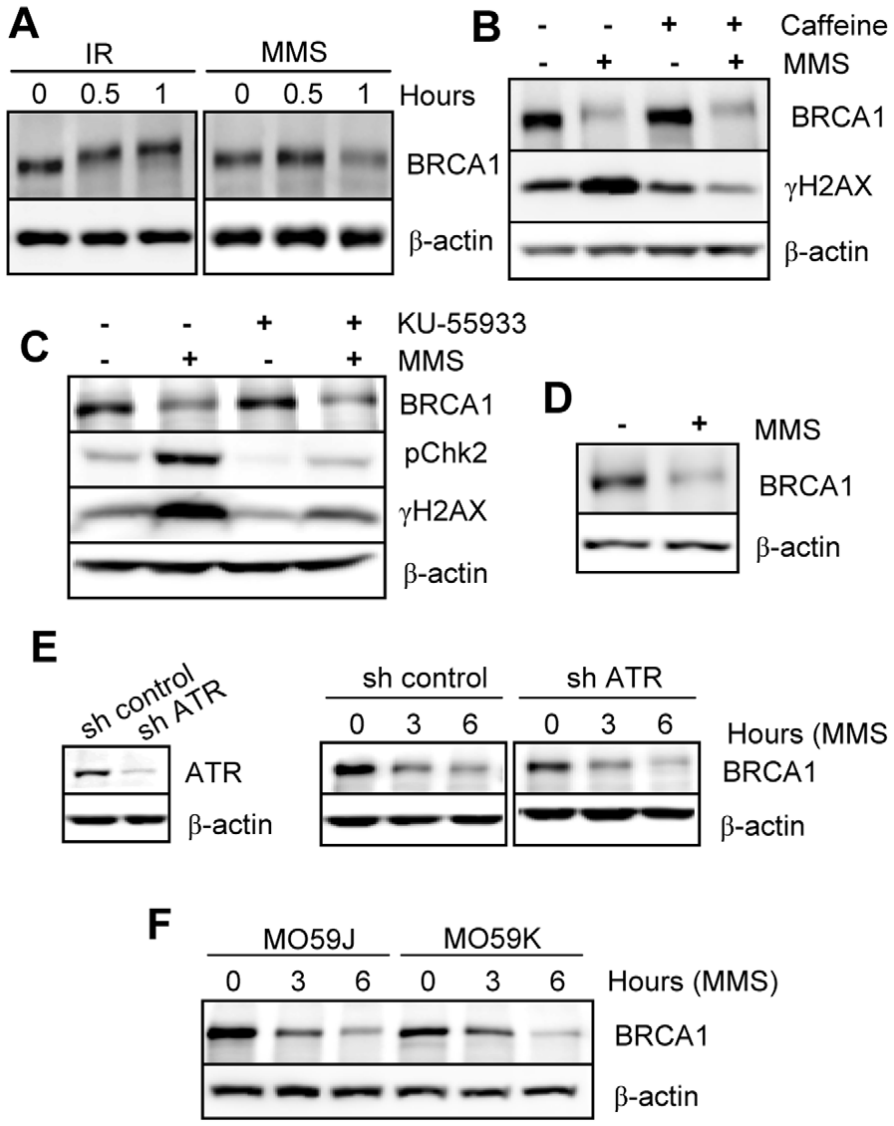
The DNA damage-activated PIKKs family members ATM, ATR and DNA-PK are not required for downregulation of BRCA1. A) Immunoblotting detection of BRCA1 in HeLa cells treated with 10 Gy IR or 200 µM MMS. B) BRCA1 downregulation is not blocked by caffeine. Immunoblotting detection of BRCA1 in HeLa cells pre-treated with 10 mM caffeine for 30 min prior to 200 µM MMS treatment for 6 hrs. C) The downregulation of BRCA1 is not prevented by the ATM inhibitor (KU-55933). Immunoblotting detection of BRCA1 in HeLa cells pre-treated with 10 µM KU-55933 for 30 min prior to 200 µM MMS treatment for 6 hrs. γH2AX and pChk2 detection were used as controls to confirm inhibition of ATM and/or ATR kinases. D) BRCA1 is downregulated in ATM-deficient human fibroblasts. Cells were treated with 200 µM MMS treatment for 6 hrs and harvested for immunoblotting. E) Depletion of ATR by RNAi does not prevent BRCA1 downregulation by MMS. *Left*, immunodetection of ATR following shRNA constructs transfection and puromycin selection. *Right*, ATR-depleted cells were treated with 200 µM MMS and harvested at the indicated times for immunoblotting. F) BRCA1 is downregulated in DNA-PKcs deficient cells. Glioblastoma DNA-PKcs proficient (MO59K) or deficient (MO59J) were treated with 200 µM MMS and harvested at the indicated times for immunoblotting.

ATM, ATR, and DNA-PK initiate multiple signaling cascades including the phosphorylation-mediated activation, stabilization, or degradation of various proteins that participate in coordinating the DNA damage response [35], [36]. Since BRCA1 is directly and rapidly phosphorylated by ATM and/or ATR, we evaluated the likely possibility of a link between PIKK signaling and BRCA1 downregulation during genotoxic stress. We first treated HeLa cells with IR or MMS for short time periods and analyzed BRCA1 protein. We found that while IR did not significantly affect BRCA1 protein levels, it induced a substantial shift in protein mobility strongly suggestive of phosphorylation (Fig. 4A). In contrast, MMS induced mainly downregulation of the protein with a less significant effect on protein mobility (Fig. 4A). Thus, phosphorylation is not correlated with BRCA1 degradation. Next, we used caffeine, a well-characterized inhibitor of ATM and ATR [37], and found that while MMS-induced H2AX phosphorylation is strongly inhibited, BRCA1 downregulation is unaffected (Fig. 4B). Similar conclusions could be drawn using the specific ATM inhibitor KU-55933 [38] (Fig. 4C) or ATM-deficient human fibroblasts (Fig. 4D and Fig.S5). As control for pharmacological inhibition of ATM, abrogation of Chk2 phosphorylation was evaluated and shown to be reduced (Fig. 4C). To specifically address the role of ATR, we used an shRNA construct which induces efficient knockdown of this protein (Fig. 4E, left panel). Following treatment with MMS, BRCA1 is downregulated to a similar extent in cells whether depleted or not for ATR (Fig. 4E, right panel). Finally, paired glioblastoma cell lines deficient or not in DNA-PK were employed to probe the potential requirement of the latter in BRCA1 downregulation. BRCA1 levels were decreased equally in DNA-PK deficient (MO59J) or proficient (MO59K) cells exposed to MMS, indicating that this kinase is dispensable for DNA damage-mediated downregulation of BRCA1 (Fig. 4F and Fig.S5). Finally, we investigated the involvement of the canonical mitogen-activated protein kinases (MAPKs), including extracellular signal-related kinase (ERK1/2), c-Jun N-terminal kinase (JNK1/2), and p38*α*/β kinase, which are rapidly activated by phosphorylation following exposure to genotoxic agents. These kinases in turn phosphorylate numerous downstream effectors that influence DNA damage-induced apoptosis, cell cycle checkpoints, and repair [39], [40], [41]. We found that inhibition of MAPK signaling using highly specific pharmacological inhibitors does not affect BRCA1 downregulation by MMS (Fig.S6). The overall data suggest that kinases other than PIKKs or MAPK family members, or possibly signals other than phosphorylation, are involved in signaling BRCA1 downregulation.

### Identification of BRCA1 domains required for DNA-damage induced BRCA1 downregulation

**Figure 5:**
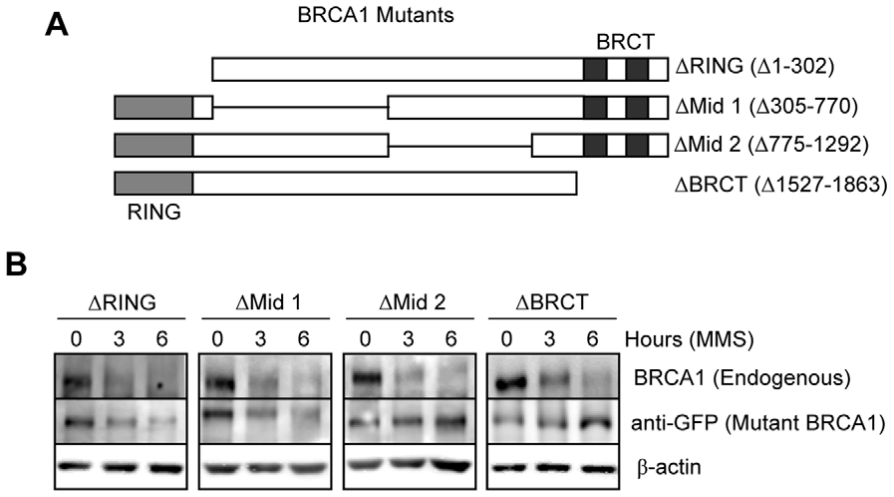
The BRCT motif, but not the Ring finger domain, is required for MMS-induced BRCA1 downregulation. A) Schematic view of the deletion constructs used in this study. B) HeLa cells were transfected with various expression constructs for BRCA1 and 2 days post-transfection, cells were treated with 200 µM MMS and harvested at the indicated times for immunoblotting to detect either endogenous BRCA1 or mutant forms using anti-BRCA1 or anti-GFP respectively. β-actin immunodetection was used as a loading control.

To provide additional insight into the mechanism of BRCA1 downregulation, we conducted functional mapping studies using expression constructs encoding BRCA1 variants lacking major functional domains (Fig. 5A). All the fragments used are expressed in HeLa cells at levels quite similar or below the levels of endogenous BRCA1. We observed that BRCA1 deleted for the N-terminal region (Δ 1-302 aa) is downregulated to a similar extent as endogenous BRCA1 following MMS treatment (Fig. 5B). This demonstrates that the ring finger is dispensable for downregulation, thereby excluding the involvement of BRCA1 ubiquitin ligase activity, and also indicates that interaction with BARD1 is not prerequisite for degradation. On the other hand, we found that BRCA1 deficient in the C-terminal region (Δ 1527–1863 aa) is completely resistant to proteasomal degradation, strongly suggesting a requirement for the BRCT domains. We also noted that BRCA1 missing the aa residues 305-770 is degraded following MMS treatment. This region contains domains required for interaction with chromatin remodeling and transcription regulators such as the SWI/SNF complex and ZBRK1 repressor [42], [43], indicating that these latter interacting partners do not play a role in BRCA1 downregulation following DNA damage. Interestingly, we found that the middle region (aa 775–1292) which encompasses the Rad51 interaction domain is essential for degradation [44]. Finally, BRCA1 lacking either the BRCT motifs or the region spanning aa 775–1292 consistently exhibited stabilization following MMS exposure, supporting the involvement of these regions in regulating BRCA1 stability following genotoxic stress.

### DNA damage-dependent downregulation of BRCA1, BARD1, BACH1 or Rad52 is mediated by the proteasome

**Figure 6:**
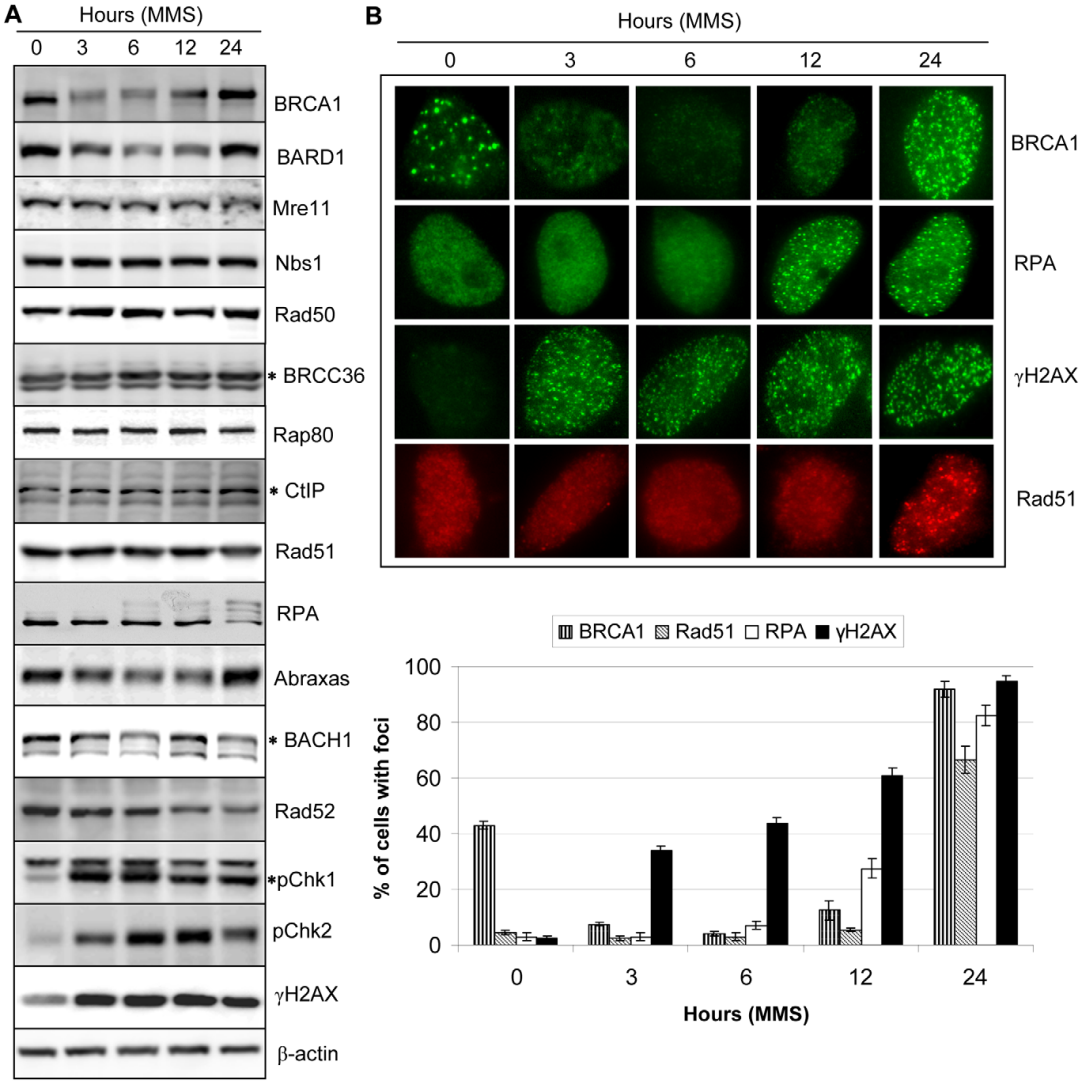
MMS induces a biphasic response of homologous recombination proteins. A) Immunodetection of various BRCA1-associated and DNA damage/repair proteins following treatment of HeLa cells with 200 µM MMS. HeLa cells were treated with 200 µM MMS and harvested at the indicated times for immunoblotting. The star indicates the specific protein band detected with a given antibody. B) Foci formation of HR proteins following MMS treatment. HeLa cells were treated with 200 µM MMS and harvested at the indicated times for immunostaining. *Bottom*, cells with more than 10 foci were counted and the data are presented as percentage of cells with foci under each condition. The values represent the average ± SD of three independent experiments.

To provide insight into the mechanism of BRCA1 downregulation, in cells treated with MMS, we investigated the stability or activation of major DNA damage response proteins known to be involved in the BRCA1 pathway (Fig. 6A). We first analyzed BARD1, the stoichiometric partner of BRCA1, and found that the former is also downregulated following MMS treatment. In addition levels of the MRN complex proteins (MRE11, NBS1 and Rad50), BRCC36, RAP80, CtIP and Rad51 all known to assemble various complexes with BRCA1/BARD1 heterodimer are not affected by MMS treatment (Fig. 6A). Moreover, no major changes of RPA protein, a marker for DNA end-resection, were observed at early time points of BRCA1/BARD1 downregulation. Strikingly however, this protein was hyperphosphorylated at the later stage of MMS exposure, as indicated by the typical shift of protein electrophoretic mobility (Fig. 6A) [45], [46], [47]. On the other hand, we did observe downregulation of Abraxas and BACH1, two other BRCT motif-interacting proteins that define the A and B complexes respectively (Fig. 6A). Interestingly, while Abraxas showed a downregulation profile similar to BRCA1 and BARD1, BACH1 exhibited a biphasic downregulation. Moreover, we found that levels of the HR protein Rad52, known to act downstream BRCA1, were significantly reduced. In addition, a slight shift in Rad52 protein gel mobility was consistently observed at the later stage of treatment (12 and 24 hours). We also observed that phosphorylation of the checkpoint kinases Chk1, Chk2, and the histone variant H2AX appear to be temporally correlated with reduction in BRCA1/BARD1/BACH1 and Rad52 protein levels (Fig. 6A). These results indicate that specific components of the HR machinery are downregulated at the early stage of the cellular response to MMS exposure and then recovered totally or partially at later times. Since RPA is hyperphosphorylated at 12 and 24 hours post-treatment, we sought to investigate the subnuclear localization of critical components of the HR pathway, i.e., BRCA1, γH2AX, RPA and Rad51. As expected from immunobloting experiments, γH2AX was strongly induced and moreover form a substantial number of γH2AX foci that, at the early stage of treatment (3–6 hours), exhibited no staining for the HR proteins BRCA1, Rad51 or RPA (Fig. 6B and Fig.S8). Interestingly, at the later stage (12–24 hours post-treatment), BRCA1, as well as RPA and Rad51, formed foci indicating DSB processing.

**Figure 7:**
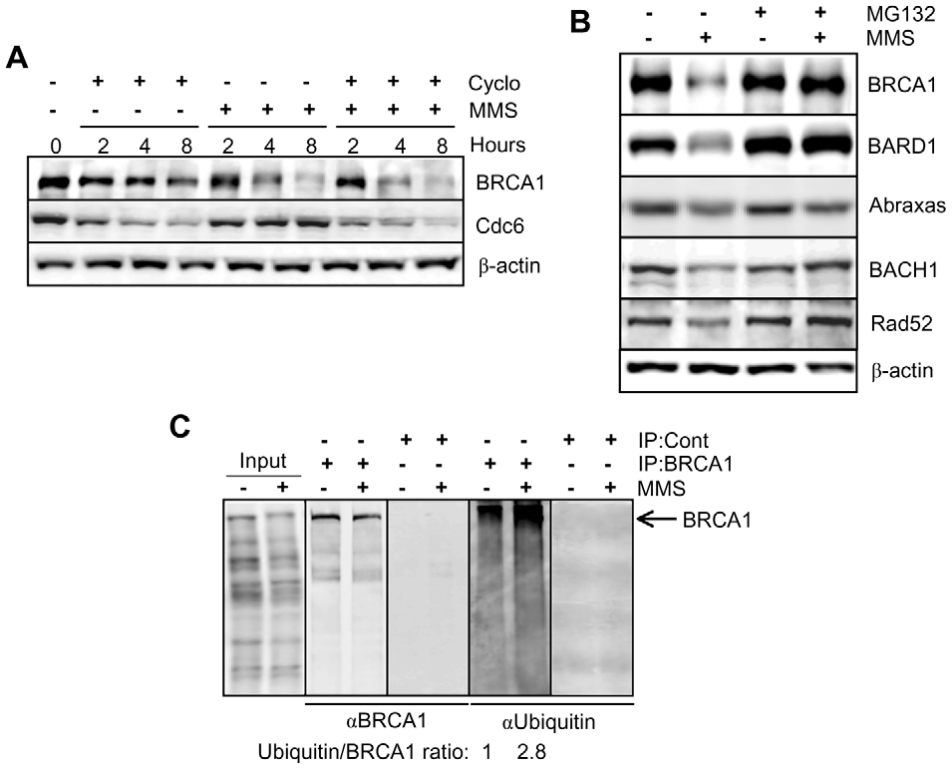
The proteasome mediates BRCA1 and BARD1 downregulation following MMS treatment. A) HeLa cells were incubated with 20 µg/ml of cycloheximide alone or with 200 µM MMS (with or without cycloheximide) and harvested at the indicated times for immunoblotting. B) HeLa cells were pre-treated with 20 µM proteasome inhibitor MG132 for 30 min and then incubated with MMS in the presence of the inhibitor and harvested at 6 hrs for immunoblotting. C) Detection of BRCA1 ubiquitination following DNA damage in HEK293T cells. Following MMS treatment for 3 hrs, cell extracts were used for immunoprecipitation with an anti-BRCA1 antibody. A non-related polyclonal antibody was used as a control. The immunoprecipitates were used for immunoblotting using anti-BRCA1 or anti-ubiquitin antibodies. Densitometry quantification was conducted on BRCA1 and ubiquitin and the ratio ubiquitin/BRCA1 is shown.

We next evaluated the possibility that MMS-induced downregulation of BRCA1 and associated partners occurs at the level of protein stability. First, a cycloheximide chase revealed that BRCA1 stability is significantly decreased in response to MMS versus cycloheximide alone, suggesting an active degradation mechanism (Fig. 7A). In contrast, the abundance of Cdc6, a protein with short half-life, is substantially decreased by treatment with cycloheximide, but not with MMS. This result prompted us to investigate the involvement of active protein degradation in regulating the stability of BRCA1 and associated partners. We found that the proteasome inhibitor MG132 completely blocks downregulation of BRCA1 (Fig. 7B). Similar results were obtained for BRCA1 following pre-treatment of HCT116 or HeLa cells with proteasome inhibitors prior to either MMS or UVC exposure (Fig.S7 A and B). Next, we analyzed additional components and found that the proteasome is also required for downregulation of BARD1, BACH1, and Rad52 in HeLa cells treated with MMS (Fig. 7B). Surprisingly, Abraxas downregulation is not blocked by MG132 suggesting that a proteasome-independent mechanism regulates levels of this protein. To demonstrate the involvement of ubiquitination *per se*, BRCA1 immunoprecipitated from either mock- or MMS-treated HEK293T cells was shown to be readily ubiquitinated following DNA damage (Fig. 7C). A densitometry quantification indicated that the ubiquitin signal is increased by ∼ 3-fold following MMS treatment. We confirmed these results in HeLa, i.e., MMS induced a 3-fold increase in BRCA1 ubiquitination (Fig.S7C). In summary, our results indicate that proteasomal-mediated degradation of BRCA1/BARD1/BACH1 and of Rad52 is a normal physiological response to DNA damaging agents that do not directly generate DSBs, and suggest the existence of a yet-to-be characterized regulatory mechanism controlling the BRCA1 pathway.

### BRCA1/BARD1 downregulation prevents their recruitment, along with Rad51, to chromatin following MMS treatment

**Figure 8:**
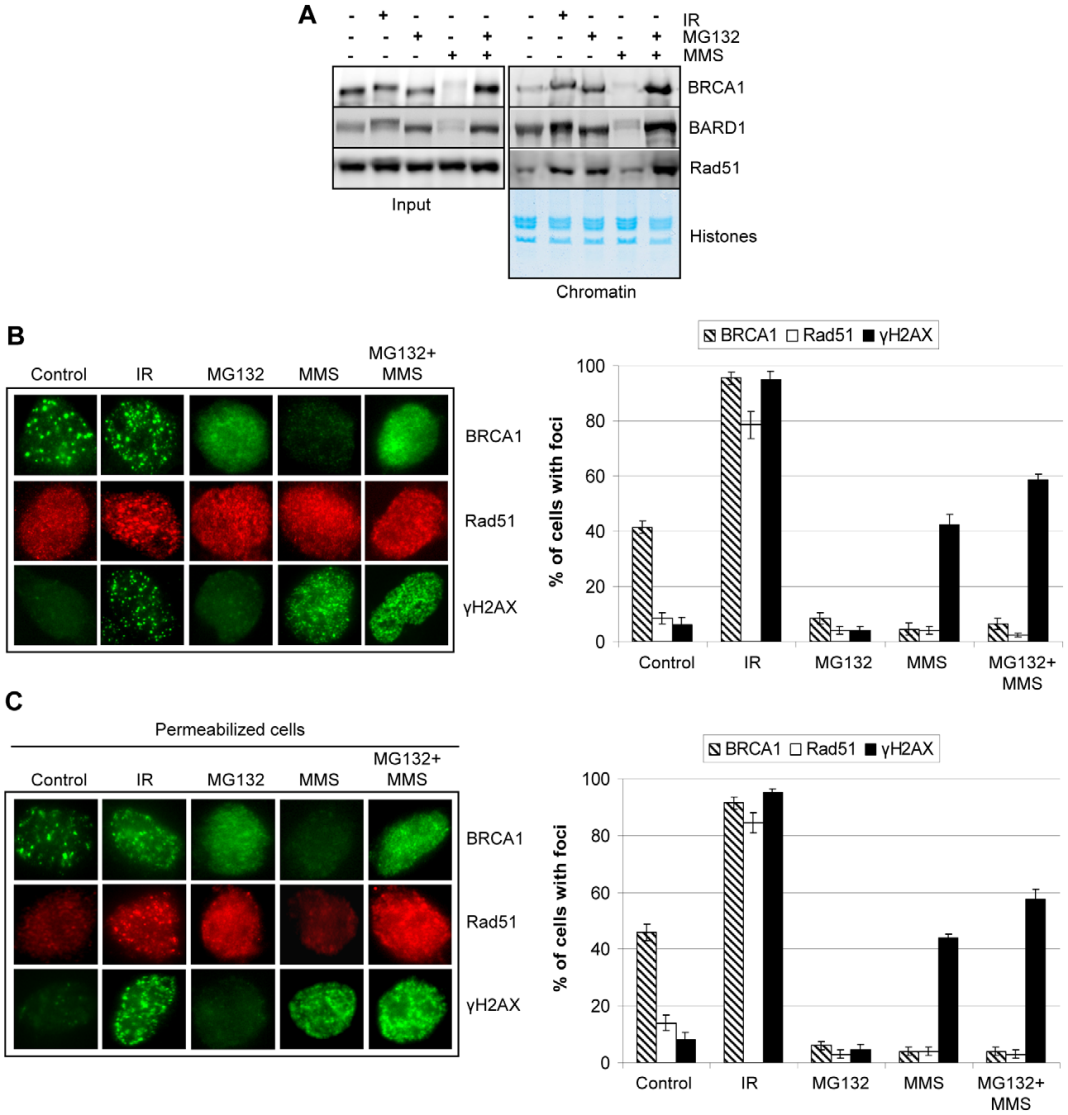
BRCA1/BARD1 downregulation prevents recruitment of these proteins along with Rad51 to chromatin following MMS treatment. A) HeLa cells were treated for 6 hrs with IR (10 Gy) or 200 µM MMS (with or without pretreatment with MG132). Chromatin from control or treated cells was prepared as described in material and methods and proteins were detected by western blotting. Histones were stained with coomassie blue to ensure equal loading. B) HeLa cells were treated as in panel A and harvested for immunostaining (*left panel*). Cells with more than 10 foci were counted and the data are presented as percentage of cells with foci under each condition (*right panel*). The values represent the average ± SD of three independent experiments. C) HeLa cells were treated as in A except that a permeabilization step was added before fixation and immunostaining (*left panel*). Cells with more than 10 foci were counted and the data are presented as percentage of cells with foci under each condition (*right panel*). The values represent the average ± SD of three independent experiments.

In response to IR-induced DSBs, phosphorylation of H2AX engenders a cascade of protein recruitment that culminates in the assembly of the BRCA1/BARD1/Rad51 HR repair complex at IRIF. The primary types of DNA damage induced by UVC and MMS are pyrimidine dimers and alkylated bases, respectively. These agents also significantly induce γH2AX (Fig. 6 and discussion). Thus we postulate that early BRCA1/BARD1 downregulation might be needed to prevent their recruitment to UV- or MMS-damaged chromatin, as this might otherwise interfere with mutagen specific-signaling events or -repair processes, i.e., nucleotide excision repair of UV-induced pyrimidine dimers or base excision repair of alkylated DNA bases. To investigate this possibility, we analyzed the recruitment of BRCA1, BARD1, and Rad51 to chromatin following inhibition of BRCA1/BARD1 downregulation using the proteasome inhibitor MG132. As control, we used IR treatment which is known to rapidly induce the assembly of BRCA1/BARD1/RAD51 on chromatin (Fig. 8A). We found, in sharp contrast to treatment with MMS or MG132 alone, that combined treatment with MMS and MG132 resulted in a highly significant recruitment of BRCA1/BARD1/RAD51 proteins to chromatin. However, it was previously shown that proteasome inhibitors block BRCA1 and Rad51 recruitment to IRIF [48]. Thus, we sought to resolve this apparent discrepancy. First, we treated HeLa cells with MG132 and found that neither BRCA1 nor Rad51 formed foci following IR thus reproducing, in our experimental setting, the previously published findings (Fig.S9). Next, we investigated the subnuclear localization of these proteins in response to MG132, MMS, or combined treatments. As control, we used IR to induce BRCA1 or Rad51 foci formation (Fig. 8B, left panel and Fig.S10). We found that BRCA1 exhibited strong but diffuse nuclear staining following treatment with either MG132 or MG132/MMS. As expected, a very low BRCA1 signal was detected in cells treated with MMS only. Rad51 staining was diffuse for all treatments except for IR, which induced its assembly at IRIF. Focus formation was observed for BRCA1/RAD51/γH2AX following IR, but only for γH2AX in the case of MMS (Fig. 8B, right panel). Altogether, these results suggest that BRCA1 and Rad51 might be loaded on chromatin in response to MG132/MMS without forming distinct foci. To further demonstrate this, we permeabilized the cells post-treatment to remove soluble cytoplasmic and nuclear proteins [49] and conducted immunostaining as above. As a control for the cell permeabilization procedure, we analyzed the nuclear protein BAP1 [50] and observed a substantial decrease of its nuclear staining (Fig.S11). We found that Rad51 and BRCA1 signals remained high with MG132/MMS, and to a lesser extent with MG132 alone, following cell permeabilization (Fig. 8C, left panel and Fig.S10). In contrast, Rad51 signal was significantly decreased in the untreated cells and following MMS, most likely due to its diffusion from the nuclei. Again, focus formation for BRCA1 and Rad51 was not observed with MG132 or MG132/MMS, as shown above for intact cells (Fig. 8C, right panel).

**Figure 9:**
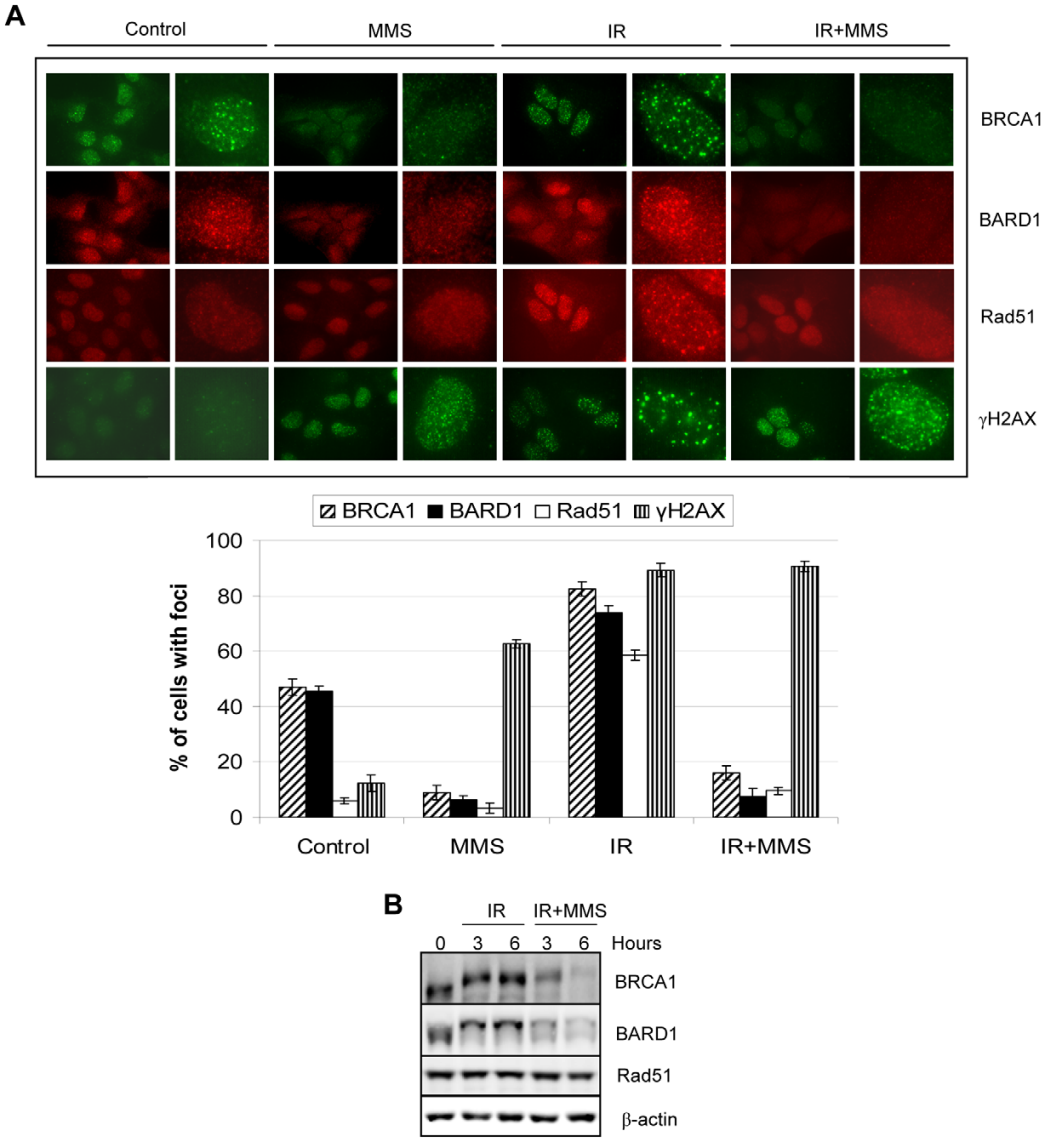
The DNA alkylating agent MMS induces the disassembly of BRCA1/BARD1/Rad51 from IRIF. A) U2OS cells were pre-treated with IR (10 Gy) for 12 hrs and then with or without 200 µM MMS for 6 hrs and harvested for immunostaining. *Bottom*, cells with more than 10 foci were counted and the data are presented as percentage of cells with foci under each condition. The values represent the average ± SD of three independent experiments. B) Immunoblotting detection of BRCA1, BARD1 and Rad51 in HeLa cells pre-treated with IR (10 Gy) for 12 hrs and then left untreated or exposed to 200 µM MMS for 3 and 6 hrs.

We next tested whether exposure to MMS might affect pre-assembled BRCA1/BARD1/RAD51 at IRIF. Cells were treated with IR in order to induce IRIF (as revealed by immunostaining for γH2AX/BRCA1/BARD1/RAD51), followed by treatment with MMS. This resulted in a dramatic decrease of BRCA1/BARD1/RAD51 foci, but not of γH2AX foci (Fig. 9A, top and bottom panels). As expected, immunoblotting indicated that although BRCA1 and BARD1 are substantially downregulated, Rad51 protein levels remain unchanged (Fig. 9B).

## Discussion

A critical role for BRCA1/BARD1 in the HR branch of DSB repair following IR exposure is now well established. However previous studies have reported conflicting results on the regulation and functionality of this heterodimer in response to genotoxic agents which induce (i) DNA adducts other than DSBs, and therefore also (ii) unique signaling pathways relative to the situation for IR (see Introduction). Here, we resolve these discrepancies by demonstrating that BRCA1 is actually downregulated rather than simply relocalized.

Indeed, the previously described dispersion of BRCA1 from constitutive foci following UVC [11], [12], or MMS exposure (this study), is associated with active degradation of the protein. However IR, which was shown to induce early dispersion of BRCA1 from constitutive foci prior to IRIF formation [11], does not induce BRCA1 downregulation. Thus, distinct signaling mechanisms are ostensibly responsible for controlling BRCA1 relocalization and/or levels during periods of genotoxic stress depending upon the nature of the DNA damage. We note that during our investigation of BRCA1 downregulation following treatment with UVC or MMS, several critical factors were taken into consideration that might account for discrepancies between previous studies and our own: (i) Total cell extracts prepared in 2% SDS, sonicated, and boiled prior to immunoblotting were used to exclude the possibility of selective extraction. (ii) Different antibodies recognizing several regions of BRCA1 were employed, thus excluding potential artifacts due to epitope masking that might be caused by post-translational modifications. (iii) Diverse human strains including primary human fibroblasts were investigated, thus controlling for potential cell-type specific responses. (iv) We showed that BRCA1 is downregulated following exposure to relatively low mutagen doses, i.e, 50 µM of MMS or 10 J/m^2^ of UVC, where within the time frame of our analysis cell viability is not compromised and apoptosis is not induced. This is important because a previous study had indicated that BRCA1 is cleaved by Caspase-3 during apoptosis, as early as 3 hrs following treatment with a very high dose of UV [27]. In addition BRCA1 downregulation is fully reversible, strongly arguing against any involvement of caspases in this early event. We also emphasize that 10 J/m^2^ of UVC is physiologically relevant as this dose induces a level of DNA photoproducts equivalent to that which can be obtained during 1 hr of exposure to natural sunlight [51], [52].

Regulation of protein stability by the ubiquitin-proteasome system is a critical determinant of protein function. Several lines of evidence presented here indicate that BRCA1 is degraded via the proteasome: (i) UVC or MMS treatment induces dramatic downregulation of BRCA1 within 2–3 hrs, and this cannot be explained by a decline of protein levels as a consequence of transcription/translation arrest since complete inhibition of protein synthesis by cycloheximide revealed that the half-life of BRCA1 is ∼4 h ([34] and this study). In addition, downregulation of BRCA1 in response to MMS treatment cannot be enhanced by pretreatment with cycloheximide, indicating that an active degradation process predominates with respect to constitutive turnover of BRCA1. (ii) Importantly, two different proteasome inhibitors (ZL3VS [53] and MG132 [54]) were used to minimize the possibility of non-specific effects. (iii) We established that BRCA1 is ubiquitinated following MMS treatment. It should be emphasized that a ubiquitination signal is also observed below that of full length BRCA1, as degradation occurs during the process of immunoprecipitation (Fig. 7C and Fig.S7C). Moreover other interacting partners of BRCA1, including BARD1 and BACH1, would also be expected to contribute to the ubiquitination signal, since these proteins are co-regulated in a proteasome-dependent manner. Building on the firm conclusions above, we decided to further elucidate novel aspects pertaining to the mechanism and significance of BRCA1/BARD1 degradation following genotoxic stress.

One preeminent event requiring consideration in the context of the current study is the rapid phosphorylation of BRCA1 by PIKK family members following genotoxic insult. Indeed the notion that PIKK signaling is required for transient proteasome-mediated downregulation of critical DNA damage responsive proteins is not without precedent. For example it was previously observed that the cyclin-dependent kinase inhibitor p21waf1 is downregulated by UVC and MMS, but not by IR, and this depends upon functional ATR kinase [55], [56]. Also the very rapid phosphorylation of BRCA1 by ATR following UV is temporally associated with BRCA1 degradation observed here. Despite these considerations, we found that inhibition of ATR, ATM, or DNA-PK does not block or enhance BRCA1 downregulation, supporting the notion that PIKK-mediated BRCA1 phosphorylation, and degradation of the protein, represent distinct signaling processes acting to control BRCA1 function. It is noteworthy that in addition to ATM, ATR, DNA-PK and MAPK, we also investigated, using specific chemical inhibitors, the potential involvement of several other kinases implicated in the DNA damage response including casein kinase 2 and cyclin-dependent kinase 2 each known to phosphorylate BRCA1 [57], [58]. Using various inhibitor concentrations, we failed to observe any interference with BRCA1 downregulation by MMS (data not shown). Taken together our data strongly suggest that phosphorylation might not be involved in triggering BRCA1 degradation. Thus, the possible involvement of other post-translational modifications or signaling events in triggering BRCA1 degradation appears quite plausible. In this respect, our work sets the stage for further studies focused on unraveling the novel signaling mechanism mediating BRCA1 downregulation following UVC- or MMS-induced DNA damage.

Interestingly we found that BRCA1 variants lacking BRCT motifs or the region spanning aa 775–1292 were not only completely resistant to degradation, but also consistently exhibited stabilization following MMS treatment. This suggests that (i) the aforementioned domains contain protein interaction motifs or sites for post-translational modifications (including ubiquitination sites) that induce degradation, and (ii) along with the engagement of active degradation, a feedback process of BRCA1 stabilization might be concomitantly induced by MMS, and this later event becomes effective only when the signaling responsible for degradation is terminated or inhibited. This feedback loop would contribute to the re-establishment of BRCA1 protein levels at the appropriate time post-genotoxic stress. Further investigations are required to address the molecular mechanism underlying this dynamic regulation of BRCA1 stability.

**Figure 10:**
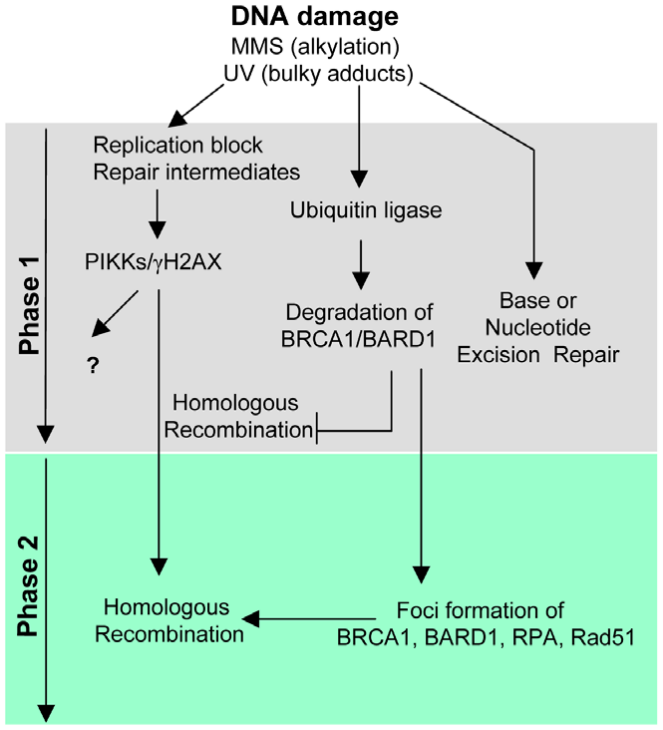
Model indicating a biphasic response of the homologous recombination pathway induced by the alkylating agent MMS. In response to MMS, human cells induce a signaling pathway that culminates in BRCA1/BARD1 downregulation. This prevents the unwanted assembly of the HR machinery at the early stage of the MMS-induced DNA damage response. At the second stage, recovery and assembly of HR proteins ensure the repair of DSBs generated by replication blocks.

It appears counterintuitive that the function of a tumor suppressor is abrogated during periods of genotoxic stress. We postulate that the biological significance of BRCA1 downregulation likely reflects a necessity to temporally coordinate DNA damage signaling and repair pathways in response to specific classes of DNA adducts. Such coordination has been proposed for other tumor suppressors involved in the maintenance of genomic integrity including the checkpoint kinase Chk1 and the DNA damage binding protein DDB2 [59], [60], [61]. Of particular note, the early transient proteasome-dependent degradation of p21waf1 mentioned above was shown to be required for efficient repair of DNA damage after UV irradiation [55], [56]. IR is well known to directly generate DSBs leading to rapid ATM/DNA-PK activation followed by phosphorylation of H2AX and subsequent DSB repair via non-homologous end-joining or HR. On the other hand neither UVC nor MMS generates DSBs as primary lesions, although both induce replication stress resulting in a delayed formation of DSBs at collapsed replication forks, which in turn induces ATM-dependent phosphorylation of H2AX [62], [63], [64]. Interestingly, it has also been reported that alkylation base damage can induce γH2AX in the complete absence of replication blockage [65]. In addition, it was clearly shown that γH2AX is upregulated by UVC treatment in the absence of DSBs and moreover associates with sites of nucleotide excision repair [66]. The exact significance of H2AX phosphorylation under such conditions is not yet clear. However since this histone modification might promote the recruitment of DSB repair proteins *per se*, it appears reasonable that critical compensatory mechanisms would be engaged to prevent the initiation of conflicting DNA damage/repair responses, i.e., in instances where no DSBs are actually induced. In fact we provide evidence that BRCA1/BARD1 degradation might prevent the untimely association of HR repair proteins with MMS-damaged chromatin, which would otherwise interfere with specific signaling events induced by alkylated DNA bases or with the execution of base excision repair. In support of this, following MMS treatment, we observed downregulation of the HR proteins Rad52, BACH1 and Abraxas, which are not immediately required to process DNA alkylation damage. For example, Rad52 interacts with Rad51, associates with single-stranded DNA ends, and promotes the annealing of complementary DNA strands [67]. Thus, its association with DNA repair intermediates generated during the processing of alkylated bases might well compromise the efficiency of base excision repair. We emphasize that downregulation of components of the HR machinery during the initial period of MMS treatment is followed by a second phase of recovery. We note that unlike BRCA1 and BARD1, Rad52 downregulation by MMS is not followed by complete recovery at 24 hours. This might suggest that only a small portion of Rad52 is needed at the later stage of MMS response, time at which HR pathway is activated. Consistent with this, a shift in Rad52 protein mobility was observed at 12 and 24 hours likely reflecting phosphorylation that might modulate its function in HR. Indeed, we observed at later stages of MMS that typical HR foci are formed and are highly enriched in BRCA1, RPA and Rad51. Importantly, foci formation was also concomitant with RPA hyperphosphorylation, a marker for DSBs processing. Clearly, the process of repair takes place after the initial phase of BRCA1/BARD1 downregulation, most likely to repair DSBs generated by replication blocking lesions. We propose a model integrating our findings, which highlight the biphasic response of HR machinery to MMS (Fig. 10).

In conclusion we have demonstrated that BRCA1/BARD1 stability and hence function is tightly regulated by ubiquitination-mediated proteasomal degradation in response to UV or MMS exposure, in a manner entirely distinct to that observed following treatment with IR. It would be extremely interesting to identify the ubiquitin ligase mediating BRCA1/BARD1 downregulation, as well as to determine how defects in this pathway affect tumor suppressor function.

## Materials and Methods

### Chemicals and plasmids

The pharmacological kinase inhibitors U0126, SP600125, and SB202190 were from Cell Signaling. Nocodazole, caffeine, cycloheximide and MG132 were from Sigma-Aldrich and KU-55933 from Calbiochem. GFP-tagged full-length BRCA1 and BRCA1 deletion mutants were provided by Dr. N. Chiba [68]. ZL_3_VS proteasome inhibitor was a generous gift of Dr. B.M. Kessler [53].

### Cell culture and DNA damage treatments

HeLa cervical cancer, U2OS osteosarcoma, HEK293 embryonic kidney, HCT116 colon carcinoma and low passage primary human foreskin fibroblasts (CCD-2056) were from ATCC, and ATM-deficient primary skin fibroblasts (HDSF, AG04405A) from the Coriell Institute. The MO59K (DNA-PK proficient) and MO59J (DNA-PK null) glioblastoma cell lines were provided by Dr. M.J. Allalunis-Turner [69]. All strains were cultured in DMEM supplemented with 10% foetal bovine serum, L-glutamine and antibiotics. Cell monolayers were washed with phosphate-buffered saline (PBS), covered with PBS, and irradiated with UVC using a crosslinker (CL-1000, VWR) at a fluency of 5 J/m^2^/s and returned to culture medium. IR exposure was performed using a cesium-137 source (Gamma Cell; Atomic Energy Canada) at a dose rate of 6.3 rad/s. Methylmethanesulfonate (MMS) (Sigma-Aldrich) was added to the culture medium at the indicated concentrations.

### Synchronization and cell cycle analysis

Primary fibroblasts we synchronized in G0/G1 by contact inhibition [34]. HeLa and U2OS cells were synchronized at the G1/S border using a thymidine double block protocol [70]. G2/M populations were obtained following 16 hours (hrs) of treatment with nocodazole (200 ng/ml) used to prevent cells from cycling. G2 cells were separated from M cells by mitotic shake off. Cell cycle analysis was carried out as described [71] using a FACScan flow cytometer fitted with CellQuestPro software (BD Biosciences).

### shRNA knockdowns

shRNA targeting ATR (TRC0000039615) was purchased from Sigma-Aldrich. The non-target control shRNA was described [72]. Cells were transfected with either shRNA and selected in medium containing puromycin for 2 days as described [71].

### Immunostaining and immunoblotting

All antibodies are described in Table S1. Western blotting using total cell extracts was performed as described [71]. The band signals were directly acquired with a LAS-3000 LCD camera coupled to MultiGauge software (Fuji, Stamford, CT, USA). Immunostaining was performed as described [71] except that the secondary antibodies Alexa Fluor 488 goat anti-mouse IgG or an Alexa Fluor 594 goat anti-rabbit IgG (Invitrogen) were used. Nuclei were counterstained with DAPI. Nuclei permeabilization was essentially conducted as previously described [49]. Fluorescence was visualized with a Leica DMRE microscope, and the data acquired using a RETIGA EX digital camera (QIMAGING) coupled with OpenLab 3.1.1 software (OpenLab).

### Immunoprecipitation

Cell extracts from control or MMS-treated cells were prepared as described [71] except that 20 mM of N-EthylMaleimide (NEM) was added to the lysis buffer. After sonication and centrifugation, lysates were incubated with anti-BRCA1 or a control IgG for 5 to 6 hrs. Immunocomplexes were recovered following 2 hrs incubation with protein G-sepharose, extensively washed with the lysis buffer, and eluted with Laemmli sample buffer for immunoblotting.

### Isolation of chromatin

Following DNA damage treatments, cells were washed with PBS and then resuspended in high-detergent containing buffer (50 mM Tris-HCl, pH 7.3; 5 mM EDTA; 150 mM KCl; 10 mM NaF, 1% Triton X-100; 1 mM phenylmethylsulfonyl fluoride (PMSF); and protease inhibitors cocktail (Sigma). Following 3 successive extractions for 15 minutes each with the same buffer, the chromatin fraction was recovered by centrifugation at 6000 g/10 min. Chromatin and total cell extracts were then used for determination of protein concentration and western blotting.

## Supporting Information

Figure S1 Immunoblotting detection of BRCA1 at various times post-UV using additional specific antibodies. (A–B) HeLa cells were treated with UVC (30 J/m^2^) and harvested at the indicated times for immunodetection with anti-BRCA1 antibodies. The monoclonal SD118 antibody which recognizes the C-terminus (A), or the polyclonal rabbit specific for the middle region (Sankaran et al., 2006) (B), were used. Immunodetection of β-actin was used as loading control.
(0.59 MB TIF)

Figure S2 Downregulation of BRCA1 with low dose of UVC. Immunoblotting detection of BRCA1 in HeLa cells following treatment with UVC (10 J/m^2^). Immunodetection of PARP-1 was used as loading control.
(0.52 MB TIF)

Figure S3 Dose-dependent downregulation of BRCA1 following MMS treatment. HEK293 Cells were harvested at the indicated time points for immunoblotting with anti-BRCA1 and anti-β-actin antibodies (left panel). The band signals were directly acquired with a LAS-3000 LCD camera (Fuji, Stamford, CT, USA) coupled to MultiGauge software (Fuji). The protein levels are relative values and are expressed as a ratio BRCA1/β-actin (right panel).
(0.65 MB TIF)

Figure S4 Downregulation of BRCA1 occurs in G2 phase. HeLa cells were synchronized in G2/M after 16 hrs exposure to nocodazole. Mitotic cells were removed by shake off and the purified G2 population was treated with 200 µM MMS for 3 hrs at various time points post-removal of nocodazole. Cell cycle analysis (left panel) and immunoblotting (right panel) were conducted at the indicated time points.
(0.63 MB TIF)

Figure S5 Immunodetection of ATM or DNA-PK in the cell lines used. Left, HeLa or ATM-deficient fibroblasts were used for immunodetection with anti-ATM antibody. Right, Immunostaining detection of DNA-PKcs in glioblastoma cell lines, proficient (MO59K) or deficient (MO59J).
(0.68 MB TIF)

Figure S6 DNA damage-activated MAPKs are not required for downregulation of BRCA1. Cells were pre-treated with 20 µM U0126, 30 µM SP600125, or 20 µM SB202190 for 30 min to inhibit signaling pathways involving ERK1/2, JNK1/2, or p38α/β, respectively (Rouget et al. 2008). Cells were then treated with 200 µM MMS and harvested after 3 hrs. Abrogation of MAPK signaling following MMS treatment was evaluated by quantification of MAPK phosphorylation using anti-phospho-ERK1/2, -JNK1/2 antibodies. The inhibition of p38α/β activity was assessed by levels of phosphorylated form of its substrate MAPKAPK2 (MK2), which can be readily distinguished from the unphosphorylated form by band shift using anti-MK2 antibody. β-actin immunodetection was used as a loading control.
(0.90 MB TIF)

Figure S7 The proteasome mediates BRCA1 downregulation in response to DNA damage. (A) HCT116 cells were pre-treated with 20 µM of the proteasome inhibitor MG132 for 30 min and then treated with 200 µM MMS in the presence of the inhibitor, and then harvested at the indicated times for immunoblotting. (B) HeLa cells were pre-treated with 20 µM of another proteasome inhibitor ZL3VS for 30 min and then treated with 30 J/m^2^ UVC in the presence of the inhibitor and harvested at the indicated times for immunoblotting. PARP-1 was used as a loading control. (C) Detection of BRCA1 ubiquitination following DNA damage in HeLa cells. Following MMS treatment for 3 hrs, cell extracts were used for immunoprecipitation with an anti-BRCA1 antibody. A non-related polyclonal antibody was used as a control. The immunoprecipitates were used for immunoblotting using anti-BRCA1 or anti-ubiquitin antibodies. Densitometric quantification was conducted on BRCA1 and ubiquitin and the ratio ubiquitin/BRCA1 is shown.
(1.11 MB TIF)

Figure S8 Immunostaining for BRCA1, Rad51, RPA or γH2AX following MMS treatment. HeLa cells were treated with 200 µM MMS and harvested for immunostaining.
(1.44 MB TIF)

Figure S9 Immunostaining for BRCA1, Rad51 and γH2AX following IR and proteasome inhibition. HeLa cells were treated for 6 hrs with IR (10 Gy) (with or without pretreatment with MG132) and harvested for immunostaining.
(1.44 MB TIF)

Figure S10 Immunostaining for BRCA1, Rad51 and γH2AX in various conditions. HeLa cells were treated for 6 hrs with IR (10 Gy) or 200 µM MMS (with or without pretreatment with MG132) and harvested for immunostaining with (top panel) or without (bottom panel) a permeabilization step.
(2.22 MB TIF)

Figure S11 Immunostaining for BAP1 following permeabilization. HeLa cells were harvested for immunostaining with (botton panel) or without (top panel) a permeabilization step.
(0.74 MB TIF)

Table S1 Antibodies used in this study.
(1.23 MB TIF)

## References

[1] Narod SA, Foulkes WD (2004). BRCA1 and BRCA2: 1994 and beyond.. Nat Rev Cancer.

[2] Huen MS, Sy SM, Chen J (2010). BRCA1 and its toolbox for the maintenance of genome integrity.. Nat Rev Mol Cell Biol.

[3] Tibbetts RS, Cortez D, Brumbaugh KM, Scully R, Livingston D (2000). Functional interactions between BRCA1 and the checkpoint kinase ATR during genotoxic stress.. Genes Dev.

[4] Xu B, O'Donnell AH, Kim ST, Kastan MB (2002). Phosphorylation of serine 1387 in Brca1 is specifically required for the Atm-mediated S-phase checkpoint after ionizing irradiation.. Cancer Res.

[5] Cortez D, Wang Y, Qin J, Elledge SJ (1999). Requirement of ATM-dependent phosphorylation of brca1 in the DNA damage response to double-strand breaks.. Science.

[6] Xu B, Kim S, Kastan MB (2001). Involvement of Brca1 in S-phase and G(2)-phase checkpoints after ionizing irradiation.. Mol Cell Biol.

[7] Lin SY, Li K, Stewart GS, Elledge SJ (2004). Human Claspin works with BRCA1 to both positively and negatively regulate cell proliferation.. Proc Natl Acad Sci U S A.

[8] O'Donovan P, Livingston DM (2010). BRCA1 and BRCA2: breast/ovarian cancer susceptibility gene products and participants in DNA double strand break repair.. Carcinogenesis.

[9] Burma S, Chen BP, Murphy M, Kurimasa A, Chen DJ (2001). ATM phosphorylates histone H2AX in response to DNA double-strand breaks.. J Biol Chem.

[10] Panier S, Durocher D (2009). Regulatory ubiquitylation in response to DNA double-strand breaks.. DNA Repair (Amst).

[11] Scully R, Chen J, Ochs RL, Keegan K, Hoekstra M (1997). Dynamic changes of BRCA1 subnuclear location and phosphorylation state are initiated by DNA damage.. Cell.

[12] Thomas JE, Smith M, Tonkinson JL, Rubinfeld B, Polakis P (1997). Induction of phosphorylation on BRCA1 during the cell cycle and after DNA damage.. Cell Growth Differ.

[13] Jin Y, Xu XL, Yang MC, Wei F, Ayi TC (1997). Cell cycle-dependent colocalization of BARD1 and BRCA1 proteins in discrete nuclear domains.. Proc Natl Acad Sci U S A.

[14] Wu X, Petrini JH, Heine WF, Weaver DT, Livingston DM (2000). Independence of R/M/N focus formation and the presence of intact BRCA1.. Science.

[15] Pageau GJ, Lawrence JB (2006). BRCA1 foci in normal S-phase nuclei are linked to interphase centromeres and replication of pericentric heterochromatin.. J Cell Biol.

[16] Gatei M, Zhou BB, Hobson K, Scott S, Young D (2001). Ataxia telangiectasia mutated (ATM) kinase and ATM and Rad3 related kinase mediate phosphorylation of Brca1 at distinct and overlapping sites. In vivo assessment using phospho-specific antibodies.. J Biol Chem.

[17] Johnson N, Cai D, Kennedy RD, Pathania S, Arora M (2009). Cdk1 participates in BRCA1-dependent S phase checkpoint control in response to DNA damage.. Mol Cell.

[18] Ray A, Mir SN, Wani G, Zhao Q, Battu A (2009). Human SNF5/INI1, a component of the human SWI/SNF chromatin remodeling complex, promotes nucleotide excision repair by influencing ATM recruitment and downstream H2AX phosphorylation.. Mol Cell Biol.

[19] Clarkin CE, Zhang H, Weber BL (2000). Kinetics of BRCA1 regulation in response to UVC radiation.. Cell Mol Life Sci.

[20] Irminger-Finger I, Leung WC, Li J, Dubois-Dauphin M, Harb J (2001). Identification of BARD1 as mediator between proapoptotic stress and p53-dependent apoptosis.. Mol Cell.

[21] Feki A, Jefford CE, Berardi P, Wu JY, Cartier L (2005). BARD1 induces apoptosis by catalysing phosphorylation of p53 by DNA-damage response kinase.. Oncogene.

[22] Yan Y, Black CP, Cao PT, Haferbier JL, Kolb RH (2008). Gamma-irradiation-induced DNA damage checkpoint activation involves feedback regulation between extracellular signal-regulated kinase 1/2 and BRCA1.. Cancer Res.

[23] Rusin M, Zajkowicz A, Butkiewicz D (2009). Resveratrol induces senescence-like growth inhibition of U-2 OS cells associated with the instability of telomeric DNA and upregulation of BRCA1.. Mech Ageing Dev.

[24] Fan S, Twu NF, Wang JA, Yuan RQ, Andres J (1998). Down-regulation of BRCA1 and BRCA2 in human ovarian cancer cells exposed to adriamycin and ultraviolet radiation.. Int J Cancer.

[25] Kranz D, Dohmesen C, Dobbelstein M (2008). BRCA1 and Tip60 determine the cellular response to ultraviolet irradiation through distinct pathways.. J Cell Biol.

[26] Brodie KM, Henderson BR (2010). Differential modulation of BRCA1 and BARD1 nuclear localisation and foci assembly by DNA damage.. Cell Signal.

[27] Zhan Q, Jin S, Ng B, Plisket J, Shangary S (2002). Caspase-3 mediated cleavage of BRCA1 during UV-induced apoptosis.. Oncogene.

[28] Yang WW, Wang ZH, Zhu Y, Yang HT (2007). E2F6 negatively regulates ultraviolet-induced apoptosis via modulation of BRCA1.. Cell Death Differ.

[29] Dizin E, Ray H, Suau F, Voeltzel T, Dalla Venezia N (2008). Caspase-dependent BRCA1 cleavage facilitates chemotherapy-induced apoptosis.. Apoptosis.

[30] Au WW, Henderson BR (2007). Identification of sequences that target BRCA1 to nuclear foci following alkylative DNA damage.. Cell Signal.

[31] Bennett CB, Westmoreland TJ, Verrier CS, Blanchette CA, Sabin TL (2008). Yeast screens identify the RNA polymerase II CTD and SPT5 as relevant targets of BRCA1 interaction.. PLoS One.

[32] Bolderson E, Richard DJ, Zhou BB, Khanna KK (2009). Recent advances in cancer therapy targeting proteins involved in DNA double-strand break repair.. Clin Cancer Res.

[33] Drew Y, Plummer R (2010). The emerging potential of poly(ADP-ribose) polymerase inhibitors in the treatment of breast cancer.. Curr Opin Obstet Gynecol.

[34] Choudhury AD, Xu H, Baer R (2004). Ubiquitination and proteasomal degradation of the BRCA1 tumor suppressor is regulated during cell cycle progression.. J Biol Chem.

[35] Abraham RT (2001). Cell cycle checkpoint signaling through the ATM and ATR kinases.. Genes Dev.

[36] Durocher D, Jackson SP (2001). DNA-PK, ATM and ATR as sensors of DNA damage: variations on a theme?. Curr Opin Cell Biol.

[37] Sarkaria JN, Busby EC, Tibbetts RS, Roos P, Taya Y (1999). Inhibition of ATM and ATR kinase activities by the radiosensitizing agent, caffeine.. Cancer Res.

[38] Hickson I, Zhao Y, Richardson CJ, Green SJ, Martin NM (2004). Identification and characterization of a novel and specific inhibitor of the ataxia-telangiectasia mutated kinase ATM.. Cancer Res.

[39] Liu ZG, Baskaran R, Lea-Chou ET, Wood LD, Chen Y (1996). Three distinct signalling responses by murine fibroblasts to genotoxic stress.. Nature.

[40] Dent P, Yacoub A, Fisher PB, Hagan MP, Grant S (2003). MAPK pathways in radiation responses.. Oncogene.

[41] Thornton TM, Rincon M (2009). Non-classical p38 map kinase functions: cell cycle checkpoints and survival.. Int J Biol Sci.

[42] Zheng L, Pan H, Li S, Flesken-Nikitin A, Chen PL (2000). Sequence-specific transcriptional corepressor function for BRCA1 through a novel zinc finger protein, ZBRK1.. Mol Cell.

[43] Bochar DA, Wang L, Beniya H, Kinev A, Xue Y (2000). BRCA1 is associated with a human SWI/SNF-related complex: linking chromatin remodeling to breast cancer.. Cell.

[44] Scully R, Chen J, Plug A, Xiao Y, Weaver D (1997). Association of BRCA1 with Rad51 in mitotic and meiotic cells.. Cell.

[45] Shao RG, Cao CX, Zhang H, Kohn KW, Wold MS (1999). Replication-mediated DNA damage by camptothecin induces phosphorylation of RPA by DNA-dependent protein kinase and dissociates RPA:DNA-PK complexes.. Embo J.

[46] Manthey KC, Opiyo S, Glanzer JG, Dimitrova D, Elliott J (2007). NBS1 mediates ATR-dependent RPA hyperphosphorylation following replication-fork stall and collapse.. J Cell Sci.

[47] Vassin VM, Anantha RW, Sokolova E, Kanner S, Borowiec JA (2009). Human RPA phosphorylation by ATR stimulates DNA synthesis and prevents ssDNA accumulation during DNA-replication stress.. J Cell Sci.

[48] Jacquemont C, Taniguchi T (2007). Proteasome function is required for DNA damage response and fanconi anemia pathway activation.. Cancer Res.

[49] Tang Y, DeFranco DB (1996). ATP-dependent release of glucocorticoid receptors from the nuclear matrix.. Mol Cell Biol.

[50] Jensen DE, Proctor M, Marquis ST, Gardner HP, Ha SI (1998). BAP1: a novel ubiquitin hydrolase which binds to the BRCA1 RING finger and enhances BRCA1-mediated cell growth suppression.. Oncogene.

[51] Kuluncsics Z, Perdiz D, Brulay E, Muel B, Sage E (1999). Wavelength dependence of ultraviolet-induced DNA damage distribution: involvement of direct or indirect mechanisms and possible artefacts.. J Photochem Photobiol B.

[52] Yoon JH, Lee CS, O'Connor TR, Yasui A, Pfeifer GP (2000). The DNA damage spectrum produced by simulated sunlight.. J Mol Biol.

[53] Kessler BM, Tortorella D, Altun M, Kisselev AF, Fiebiger E (2001). Extended peptide-based inhibitors efficiently target the proteasome and reveal overlapping specificities of the catalytic beta-subunits.. Chem Biol.

[54] Lee DH, Goldberg AL (1998). Proteasome inhibitors: valuable new tools for cell biologists.. Trends Cell Biol.

[55] Bendjennat M, Boulaire J, Jascur T, Brickner H, Barbier V (2003). UV irradiation triggers ubiquitin-dependent degradation of p21(WAF1) to promote DNA repair.. Cell.

[56] Soria G, Gottifredi V (2010). PCNA-coupled p21 degradation after DNA damage: The exception that confirms the rule?. DNA Repair (Amst).

[57] O'Brien KA, Lemke SJ, Cocke KS, Rao RN, Beckmann RP (1999). Casein kinase 2 binds to and phosphorylates BRCA1.. Biochem Biophys Res Commun.

[58] Ruffner H, Jiang W, Craig AG, Hunter T, Verma IM (1999). BRCA1 is phosphorylated at serine 1497 in vivo at a cyclin-dependent kinase 2 phosphorylation site.. Mol Cell Biol.

[59] Zhang YW, Otterness DM, Chiang GG, Xie W, Liu YC (2005). Genotoxic stress targets human Chk1 for degradation by the ubiquitin-proteasome pathway.. Mol Cell.

[60] Rapic-Otrin V, McLenigan MP, Bisi DC, Gonzalez M, Levine AS (2002). Sequential binding of UV DNA damage binding factor and degradation of the p48 subunit as early events after UV irradiation.. Nucleic Acids Res.

[61] Fitch ME, Cross IV, Turner SJ, Adimoolam S, Lin CX (2003). The DDB2 nucleotide excision repair gene product p48 enhances global genomic repair in p53 deficient human fibroblasts.. DNA Repair (Amst).

[62] Ward IM, Chen J (2001). Histone H2AX is phosphorylated in an ATR-dependent manner in response to replicational stress.. J Biol Chem.

[63] Ward IM, Minn K, Chen J (2004). UV-induced ataxia-telangiectasia-mutated and Rad3-related (ATR) activation requires replication stress.. J Biol Chem.

[64] Staszewski O, Nikolova T, Kaina B (2008). Kinetics of gamma-H2AX focus formation upon treatment of cells with UV light and alkylating agents.. Environ Mol Mutagen.

[65] Liu JS, Kuo SR, Melendy T (2003). Comparison of checkpoint responses triggered by DNA polymerase inhibition versus DNA damaging agents.. Mutat Res.

[66] Marti TM, Hefner E, Feeney L, Natale V, Cleaver JE (2006). H2AX phosphorylation within the G1 phase after UV irradiation depends on nucleotide excision repair and not DNA double-strand breaks.. Proc Natl Acad Sci U S A.

[67] Symington LS (2002). Role of RAD52 epistasis group genes in homologous recombination and double-strand break repair.. Microbiol Mol Biol Rev.

[68] Wei L, Lan L, Hong Z, Yasui A, Ishioka C (2008). Rapid recruitment of BRCA1 to DNA double-strand breaks is dependent on its association with Ku80.. Mol Cell Biol.

[69] Anderson CW, Dunn JJ, Freimuth PI, Galloway AM, Allalunis-Turner MJ (2001). Frameshift mutation in PRKDC, the gene for DNA-PKcs, in the DNA repair-defective, human, glioma-derived cell line M059J.. Radiat Res.

[70] Harper JV (2005). Synchronization of cell populations in G1/S and G2/M phases of the cell cycle.. Methods Mol Biol.

[71] Affar EB, Gay F, Shi Y, Liu H, Huarte M (2006). Essential dosage-dependent functions of the transcription factor Yin Yang 1 in late embryonic development and cell cycle progression.. Mol Cell Biol.

[72] Sui G, Affar el B, Shi Y, Brignone C, Wall NR (2004). Yin Yang 1 is a negative regulator of p53.. Cell.

