# Supplementary figures and images for: PI 3 Kinase Related Kinases-Independent Proteolysis of BRCA1 Regulates Rad51 Recruitment during Genotoxic Stress in Human Cells
Source: PLoS One. 2010 Nov 17;5(11):e14027. doi: 10.1371/journal.pone.0014027 (PMC2984446; doi:10.1371/journal.pone.0014027)

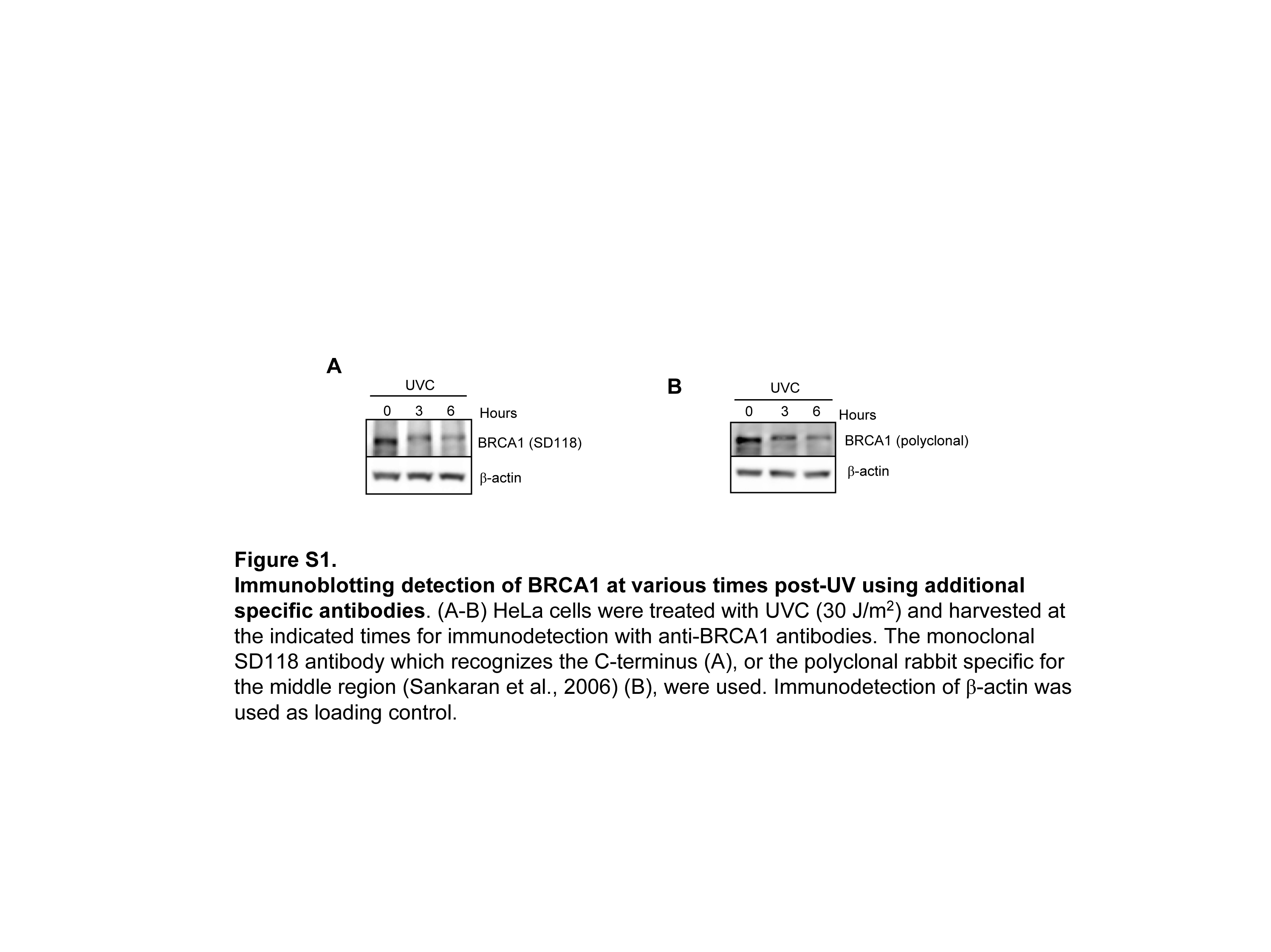

Supplement: Figure S1 — Immunoblotting detection of BRCA1 at various times post-UV using additional specific antibodies. (A–B) HeLa cells were treated with UVC (30 J/m2) and harvested at the indicated times for immunodetection with anti-BRCA1 antibodies. The monoclonal SD118 antibody which recognizes the C-terminus (A), or the polyclonal rabbit specific for the middle region (Sankaran et al., 2006) (B), were used. Immunodetection of β-actin was used as loading control. (0.59 MB TIF) [file pone.0014027.s001.tif]

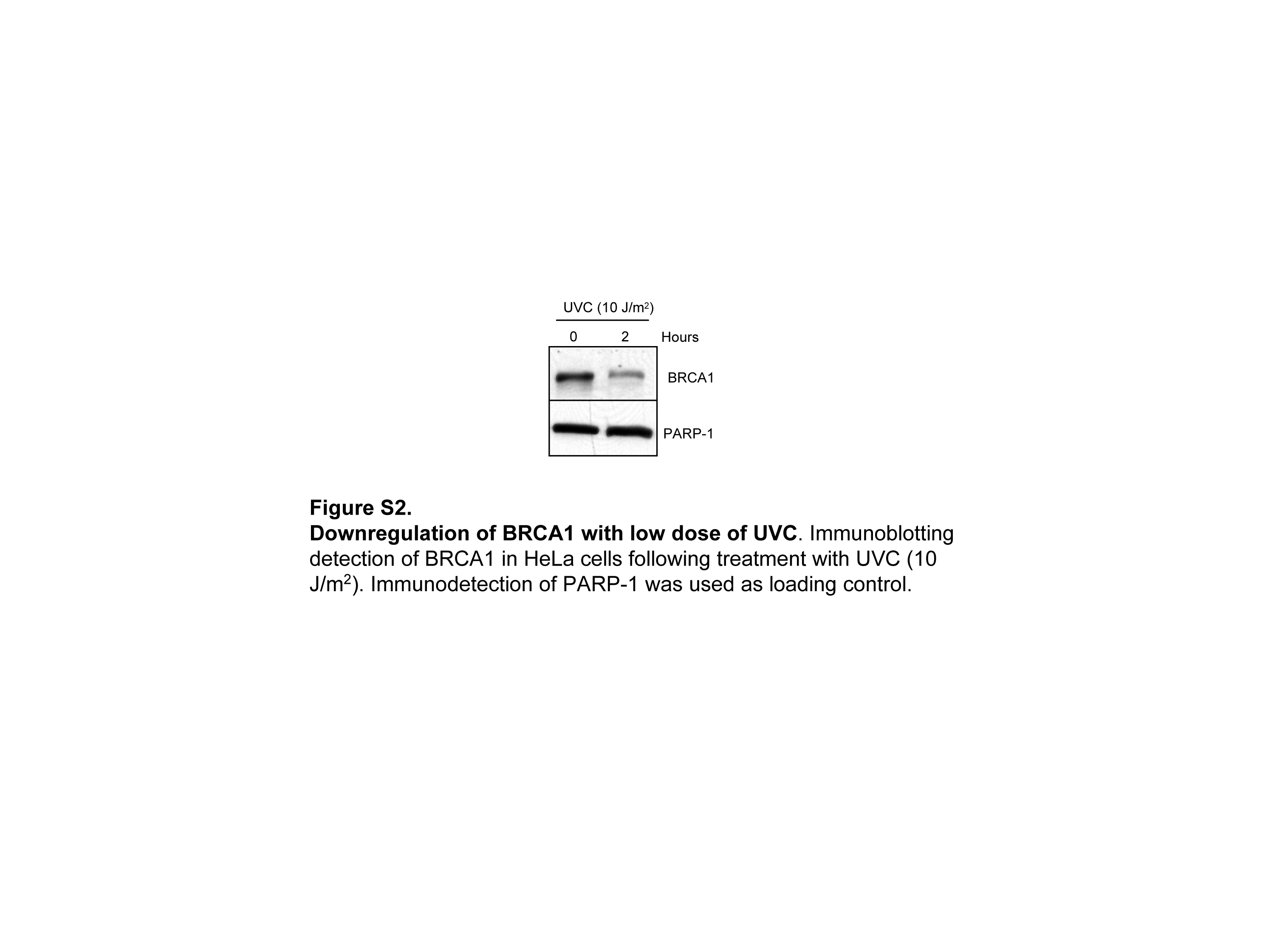

Supplement: Figure S2 — Downregulation of BRCA1 with low dose of UVC. Immunoblotting detection of BRCA1 in HeLa cells following treatment with UVC (10 J/m2). Immunodetection of PARP-1 was used as loading control. (0.52 MB TIF) [file pone.0014027.s002.tif]

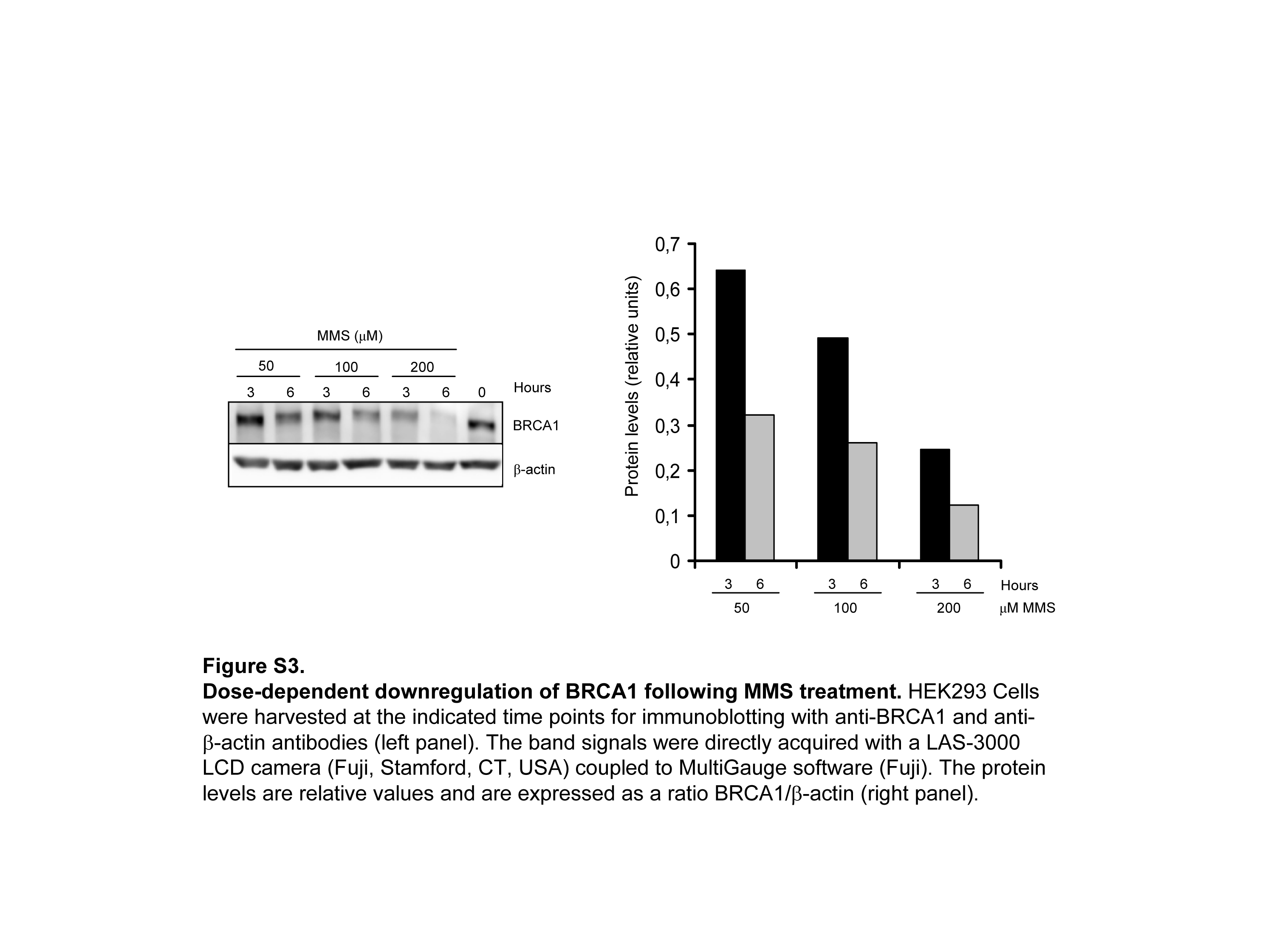

Supplement: Figure S3 — Dose-dependent downregulation of BRCA1 following MMS treatment. HEK293 Cells were harvested at the indicated time points for immunoblotting with anti-BRCA1 and anti-β-actin antibodies (left panel). The band signals were directly acquired with a LAS-3000 LCD camera (Fuji, Stamford, CT, USA) coupled to MultiGauge software (Fuji). The protein levels are relative values and are expressed as a ratio BRCA1/β-actin (right panel). (0.65 MB TIF) [file pone.0014027.s003.tif]

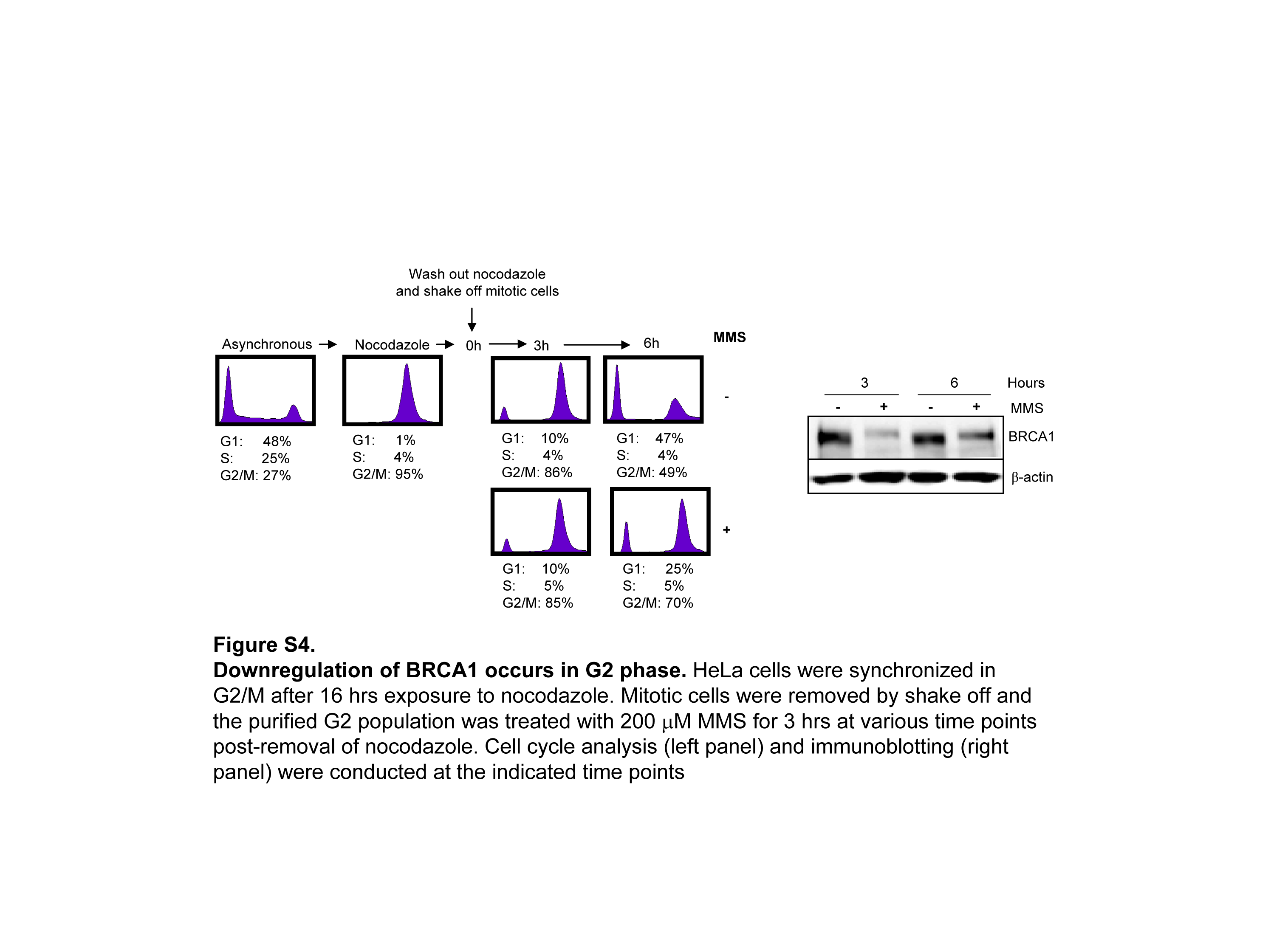

Supplement: Figure S4 — Downregulation of BRCA1 occurs in G2 phase. HeLa cells were synchronized in G2/M after 16 hrs exposure to nocodazole. Mitotic cells were removed by shake off and the purified G2 population was treated with 200 µM MMS for 3 hrs at various time points post-removal of nocodazole. Cell cycle analysis (left panel) and immunoblotting (right panel) were conducted at the indicated time points. (0.63 MB TIF) [file pone.0014027.s004.tif]

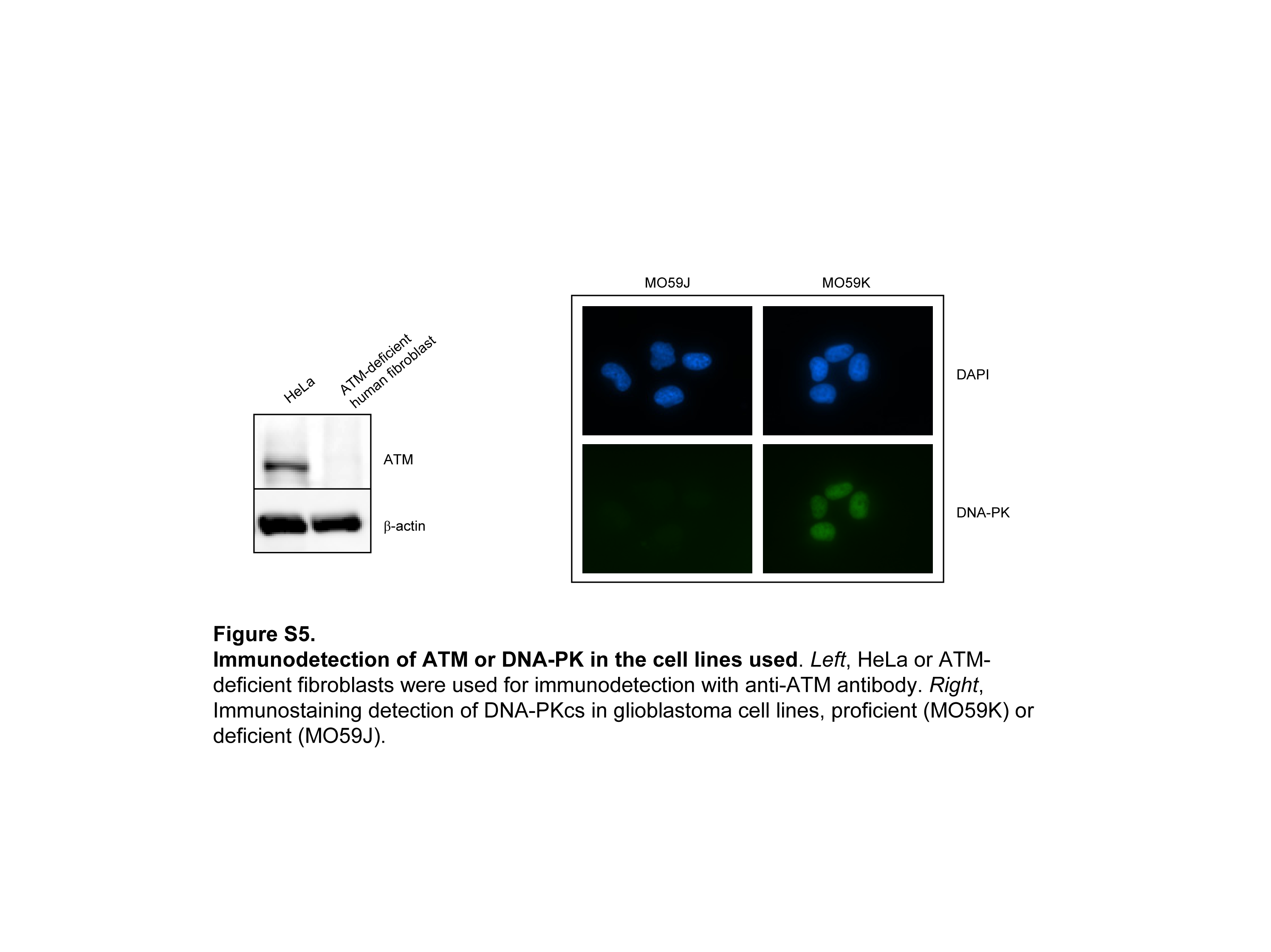

Supplement: Figure S5 — Immunodetection of ATM or DNA-PK in the cell lines used. Left, HeLa or ATM-deficient fibroblasts were used for immunodetection with anti-ATM antibody. Right, Immunostaining detection of DNA-PKcs in glioblastoma cell lines, proficient (MO59K) or deficient (MO59J). (0.68 MB TIF) [file pone.0014027.s005.tif]

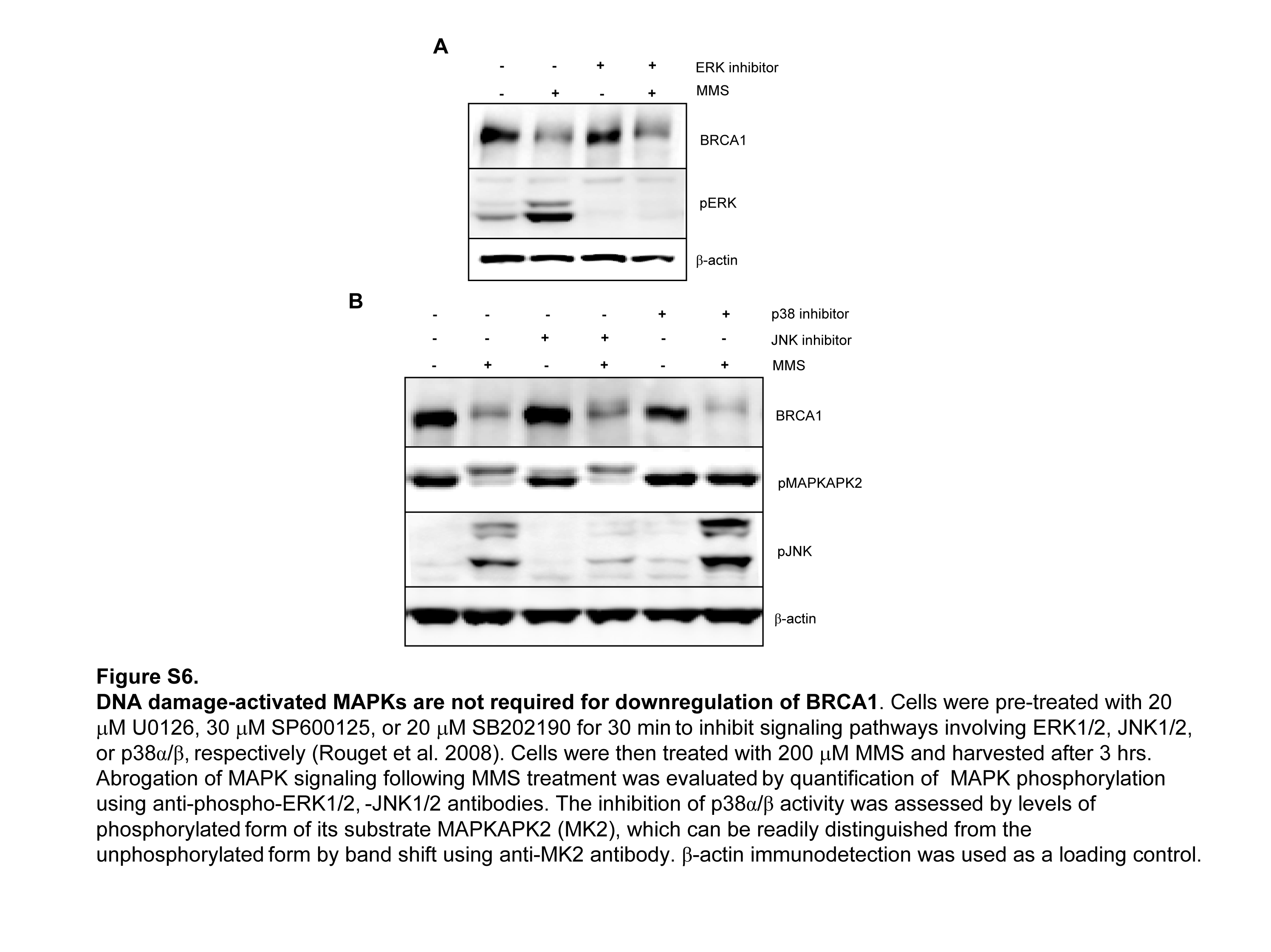

Supplement: Figure S6 — DNA damage-activated MAPKs are not required for downregulation of BRCA1. Cells were pre-treated with 20 µM U0126, 30 µM SP600125, or 20 µM SB202190 for 30 min to inhibit signaling pathways involving ERK1/2, JNK1/2, or p38α/β, respectively (Rouget et al. 2008). Cells were then treated with 200 µM MMS and harvested after 3 hrs. Abrogation of MAPK signaling following MMS treatment was evaluated by quantification of MAPK phosphorylation using anti-phospho-ERK1/2, -JNK1/2 antibodies. The inhibition of p38α/β activity was assessed by levels of phosphorylated form of its substrate MAPKAPK2 (MK2), which can be readily distinguished from the unphosphorylated form by band shift using anti-MK2 antibody. β-actin immunodetection was used as a loading control. (0.90 MB TIF) [file pone.0014027.s006.tif]

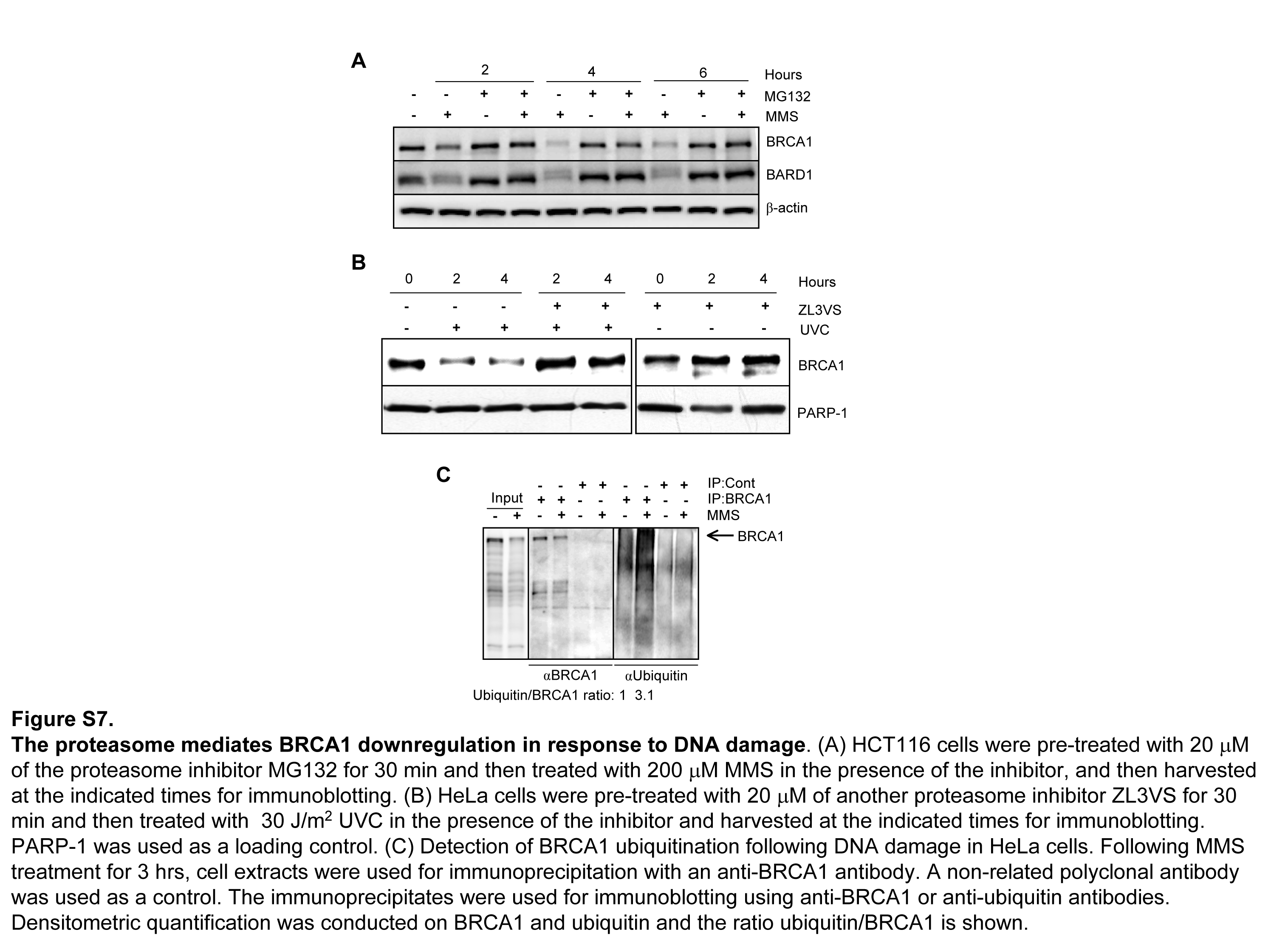

Supplement: Figure S7 — The proteasome mediates BRCA1 downregulation in response to DNA damage. (A) HCT116 cells were pre-treated with 20 µM of the proteasome inhibitor MG132 for 30 min and then treated with 200 µM MMS in the presence of the inhibitor, and then harvested at the indicated times for immunoblotting. (B) HeLa cells were pre-treated with 20 µM of another proteasome inhibitor ZL3VS for 30 min and then treated with 30 J/m2 UVC in the presence of the inhibitor and harvested at the indicated times for immunoblotting. PARP-1 was used as a loading control. (C) Detection of BRCA1 ubiquitination following DNA damage in HeLa cells. Following MMS treatment for 3 hrs, cell extracts were used for immunoprecipitation with an anti-BRCA1 antibody. A non-related polyclonal antibody was used as a control. The immunoprecipitates were used for immunoblotting using anti-BRCA1 or anti-ubiquitin antibodies. Densitometric quantification was conducted on BRCA1 and ubiquitin and the ratio ubiquitin/BRCA1 is shown. (1.11 MB TIF) [file pone.0014027.s007.tif]

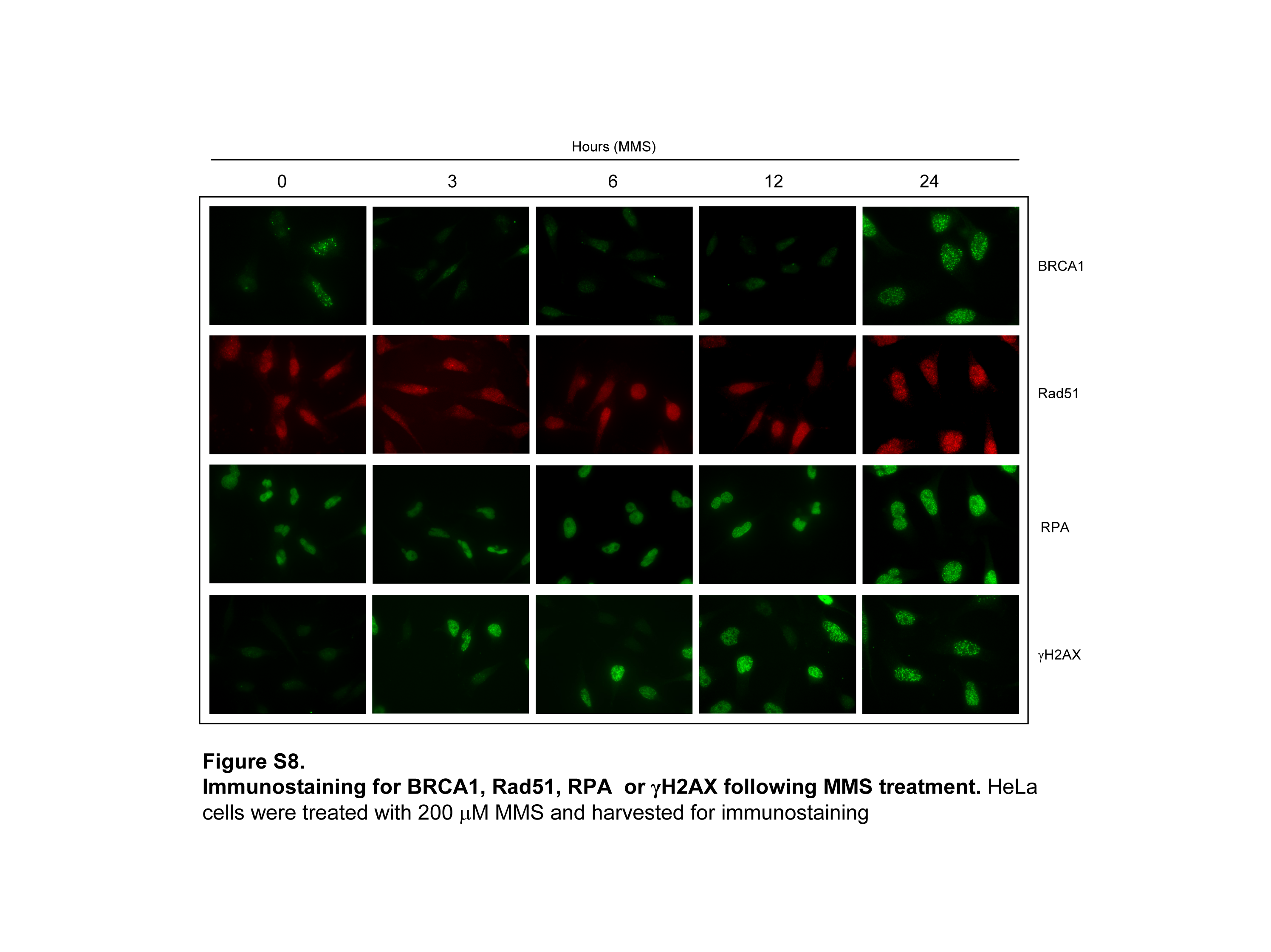

Supplement: Figure S8 — Immunostaining for BRCA1, Rad51, RPA or γH2AX following MMS treatment. HeLa cells were treated with 200 µM MMS and harvested for immunostaining. (1.44 MB TIF) [file pone.0014027.s008.tif]

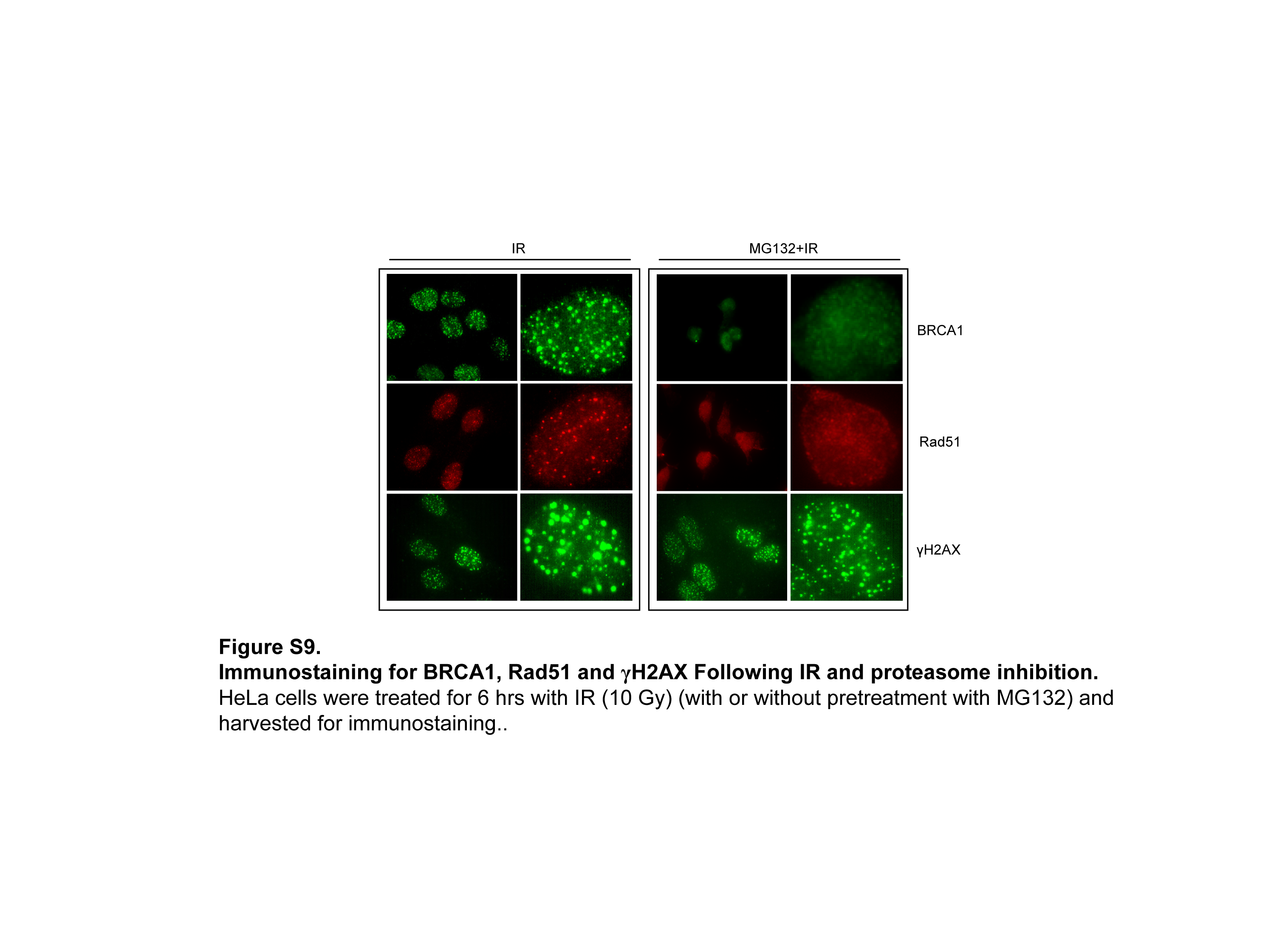

Supplement: Figure S9 — Immunostaining for BRCA1, Rad51 and γH2AX following IR and proteasome inhibition. HeLa cells were treated for 6 hrs with IR (10 Gy) (with or without pretreatment with MG132) and harvested for immunostaining. (1.44 MB TIF) [file pone.0014027.s009.tif]

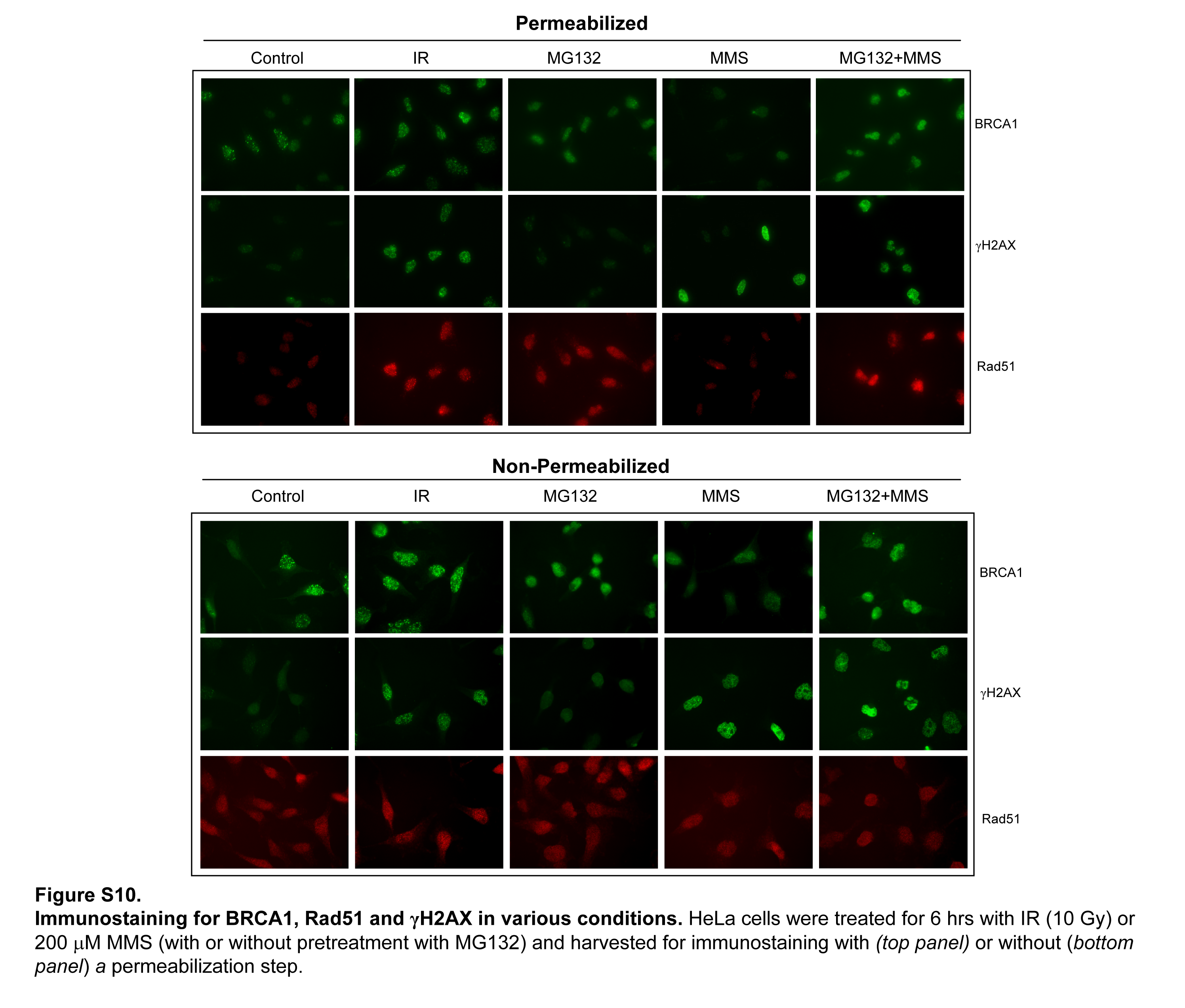

Supplement: Figure S10 — Immunostaining for BRCA1, Rad51 and γH2AX in various conditions. HeLa cells were treated for 6 hrs with IR (10 Gy) or 200 µM MMS (with or without pretreatment with MG132) and harvested for immunostaining with (top panel) or without (bottom panel) a permeabilization step. (2.22 MB TIF) [file pone.0014027.s010.tif]

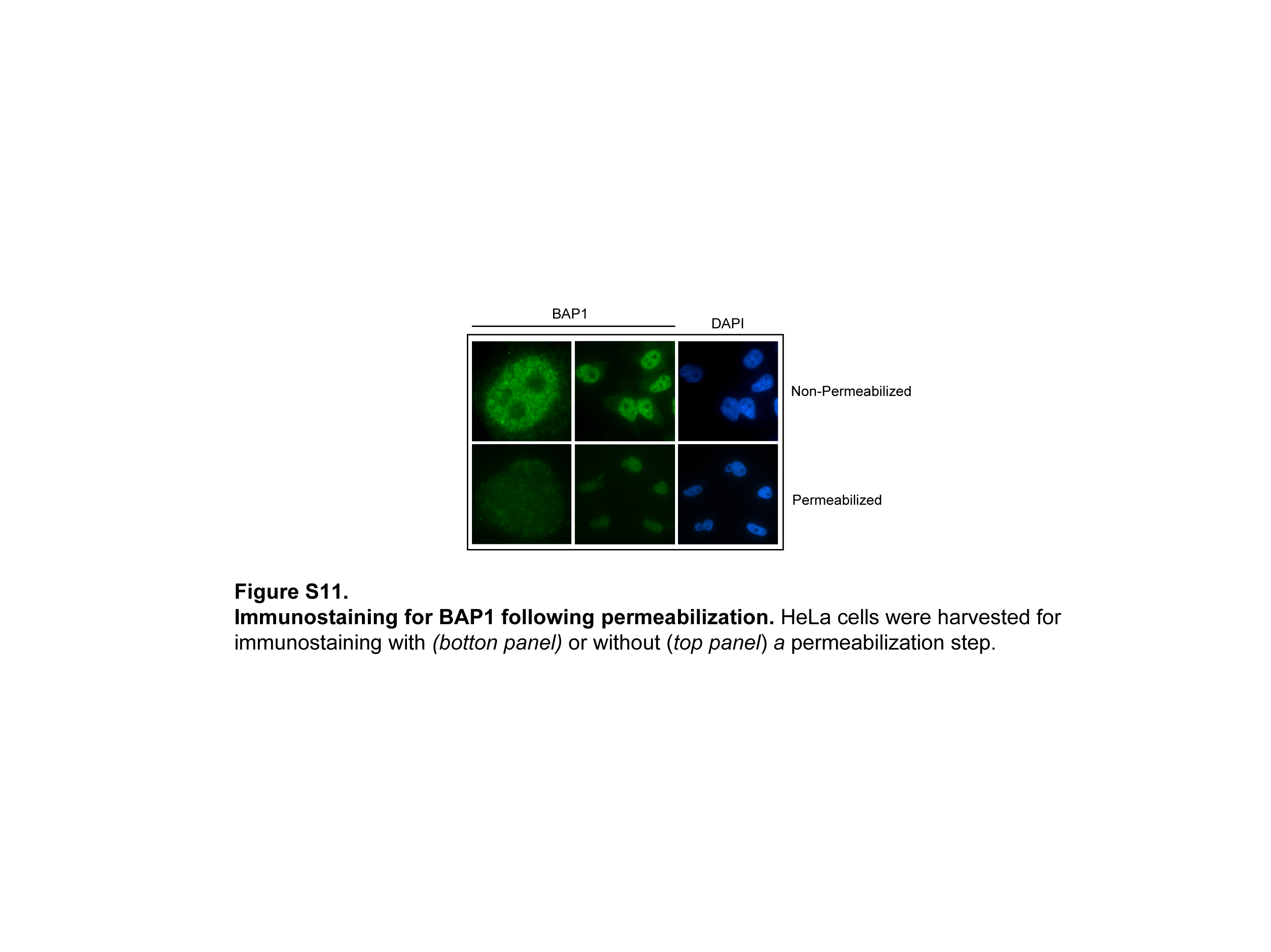

Supplement: Figure S11 — Immunostaining for BAP1 following permeabilization. HeLa cells were harvested for immunostaining with (botton panel) or without (top panel) a permeabilization step. (0.74 MB TIF) [file pone.0014027.s011.tif]

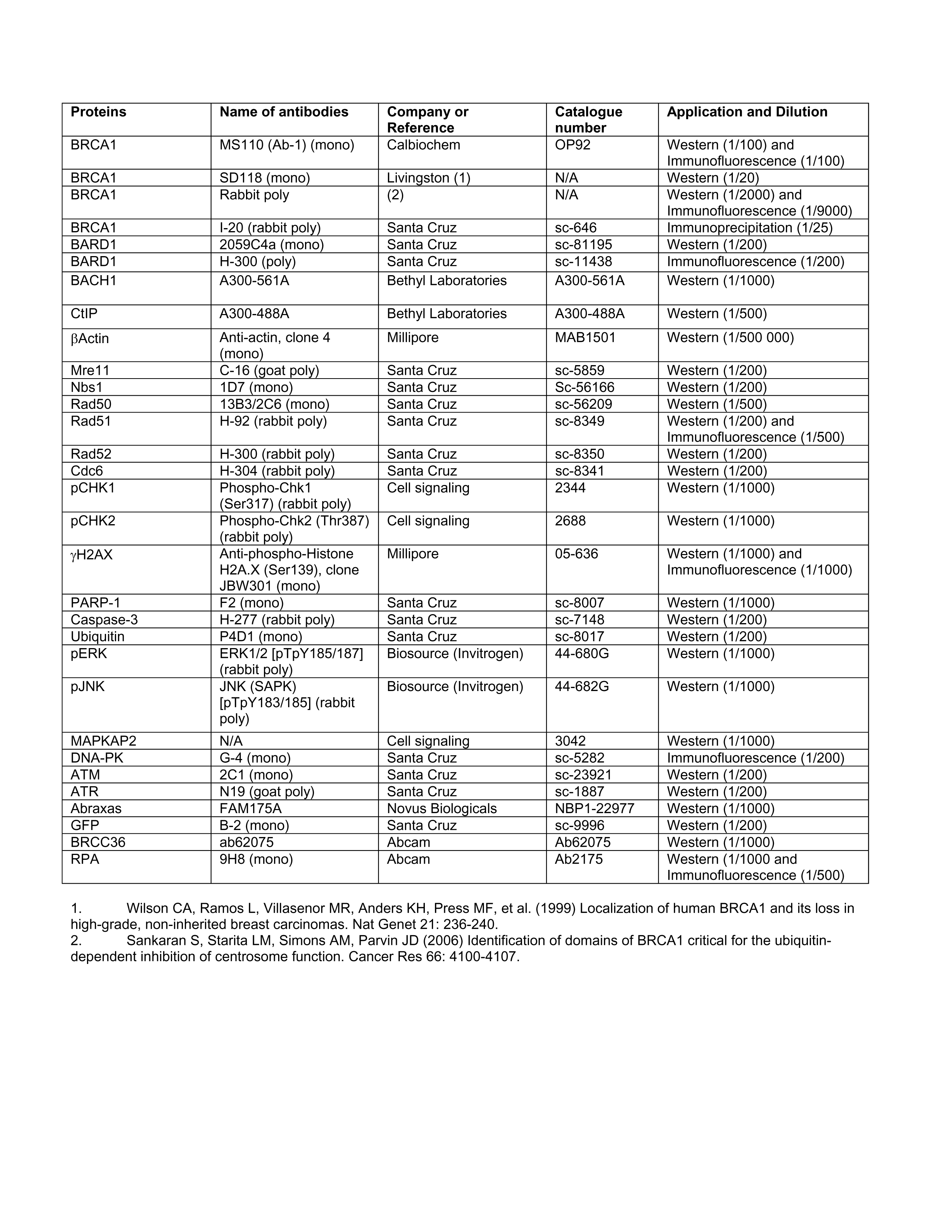

Supplement: Table S1 — Antibodies used in this study. (1.23 MB TIF) [file pone.0014027.s012.tif]
